# Visualizing differences in phylogenetic information content of alignments and distinction of three classes of long-branch effects

**DOI:** 10.1186/1471-2148-7-147

**Published:** 2007-08-28

**Authors:** Johann Wolfgang Wägele, Christoph Mayer

**Affiliations:** 1 Zoologisches Forschungsmuseum Alexander Koenig, 53113 Bonn, Germany; 2 Lehrstuhl Spezielle Zoologie, Faculty of Biology, University Bochum, 44780 Bochum, Germany

## Abstract

**Background:**

Published molecular phylogenies are usually based on data whose quality has not been explored prior to tree inference. This leads to errors because trees obtained with conventional methods suppress conflicting evidence, and because support values may be high even if there is no distinct phylogenetic signal. Tools that allow an a priori examination of data quality are rarely applied.

**Results:**

Using data from published molecular analyses on the phylogeny of crustaceans it is shown that tree topologies and popular support values do not show existing differences in data quality. To visualize variations in signal distinctness, we use network analyses based on split decomposition and split support spectra. Both methods show the same differences in data quality and the same clade-supporting patterns. Both methods are useful to discover long-branch effects.

We discern three classes of long branch effects. Class I effects consist of attraction of terminal taxa caused by symplesiomorphies, which results in a false monophyly of paraphyletic groups. Addition of carefully selected taxa can fix this effect. Class II effects are caused by drastic signal erosion. Long branches affected by this phenomenon usually slip down the tree to form false clades that in reality are polyphyletic. To recover the correct phylogeny, more conservative genes must be used. Class III effects consist of attraction due to accumulated chance similarities or convergent character states. This sort of noise can be reduced by selecting less variable portions of the data set, avoiding biases, and adding slower genes.

**Conclusion:**

To increase confidence in molecular phylogenies an exploratory analysis of the signal to noise ratio can be conducted with split decomposition methods. If long-branch effects are detected, it is necessary to discern between three classes of effects to find the best approach for an improvement of the raw data.

## Background

Assuming a reliable alignment is available, phylogenetic tree topologies inferred from molecular data usually are regarded to be informative, if support for clades as estimated with bootstrapping or jackknifing methods or with Bayesian approaches is high and if at the same time a systematic bias can be excluded [e.g. [1-12]]. Even though it is well known by scientists interested in theory that bootstrap values give no indication of whether there is a systematic problem within the data set [13], that Bayesian support values may be too optimistic [14], and that a bias may cause convergence to an incorrect tree, most biologists still rely on bootstrapping or on Bayesian support values. However, "bootstrap support of 100% is not enough, the tree must also be correct" [15].

Phylogeny inference is an inductive science that depends on sampling of empirical data. Usually, in natural sciences the quality of empirical data must be evaluated to detect sampling errors and differences in quality of sampled data before any conclusions are derived.

However, in molecular systematics raw data are generally not tested for their suitability to detect the phylogenetic history of organisms *before *tree construction. All popular methods used to assess the reliability of an analysis compare the fit between results and data (e.g. via bootstrapping). This approach can be misleading. Clades may get a good support whenever parts of the topology are based on compatible patterns in an alignment, even if these patterns are not traces of the real phylogeny. It also may happen that several contradicting phylogenies get high support values depending on the method used [15]. This is not only a problem of model selection but also of the amount and quality of available information.

A number of implausible phylogenies have been published by very confident authors who proclaimed the discovery of surprising relationships that clearly contradict most of the available background knowledge. Examples are the improbable Marsupionta hypothesis (monophyly of {Monotremata, Marsupialia}) that was based on analyses of complete mitochondrial genomes [16-18], which later was refuted several times due to morphological evidence and because of results obtained with alignments of nuclear genes [19-23]. According to Phillips and Penny [23], the Marsupionta clade can be explained by parallel shifts in base frequencies in mitochondrial genomes of Monotremata and Marsupialia. Another case is the prominently published mollusc phylogeny with polyphyletic snails and mussels [24], which also is extremely improbable in view of the bulk of information available to zoologists [e.g. [25-29]]. Until now, nobody has proposed that the bauplan of snails or mussels evolved independently several times and that e.g. characters shared by mussels are convergences. In both examples the authors did not check whether their alignments contain contradicting signal-like patterns. Many other examples exist, some are analysed in the following. Even though tools have been published that allow an *a priori *check of data quality [30-36], it seems that most biologists are not aware of the necessity to ask whether their data are suitable for a phylogenetic analysis or not.

Real data sets always contain conflicting information. The processes producing conflicts are well understood. Excluding cases of horizontal gene transfer, the structure of the data may not be tree-like due to lack of historical signal or due to presence of non-historical signals and stochastic errors. Remember that historical signals are always real homologies, and that only those homologies that evolved on the stem-lineage of a real clade, the so-called apomorphies, can substantiate the existence (monophyly) of this clade [37]. Apomorphies shared by different taxa are called synapomorphies.

It may be that multiple substitutions destroy synapomorphies (process of signal erosion), homoplasies can accumulate along "long branches". Substitution processes not only produce phylogenetic signal (apomorphic character states) but also chance similarities that may attract distantly related clades in a topology. If species radiated quickly or if stem-lineages are short, apomorphies that evolved in stem-lineages may be rare and chance similarities that evolved later can dominate in the form of signal-like patterns [38-41]. It has been shown in simulations and it is often claimed that substitution models can correct some of these effects [42-47], but there is no way to discover without background knowledge whether the phylogeny is plausible and whether the selected model conveniently corrects for misleading events. With unrealistic models likelihood methods can converge on the wrong tree [48-50].

Under an ideal model of DNA evolution, a random Markov process would produce randomly distributed analogies. As long as some phylogenetic signal is conserved, this random noise in the data can be corrected with an appropriate substitution model. However, in nature selection by environmental parameters and developmental constraints produce non-random patterns. Even assuming that gene conversion between paralogous genes and lateral (horizontal) transfer of genes between species are rare, effects of unknown population bottle necks and other unknown factors influencing substitution rates in different sequence regions and different lineages can produce non-phylogenetic signals that can not always be recognized. Therefore, any phylogenetic analyses should begin with an exploratory assessment of the quality of the data set.

Our concept for the terms signal and noise must be explained here. For the purpose of phylogenetic analyses, a signal is an identifiable trace left by phylogeny in heritable characters, in our case, in genes. Signal consists of character states which are homologous, which can be identified due to their identity or which can be derived from each other with an appropriate model of character evolution. For our discussion, noise is any modification of sequences that destroys the true signal or that produces false signals. Noise can consist of random data [40], it can be the effect of randomly distributed substitutions, but also of convergence triggered by selective forces and affecting base composition, site variability and covariation, or evolutionary rates. Presence of paralogous sequences can also introduce noise in form of conflicting signals. Different types of false signals, classified by us as noise, have also been named compositional signals, heterotachous signals, or rate signals [51].

For any phylogenetic analysis two different sets of questions must be discerned. There are questions concerning data quality: How informative is the data set? Does it contain compatible signal-like patterns or do contradicting signals dominate? Is there enough phylogenetic signal to infer the correct substitution model? Is it possible to discern signal and noise? And there are questions concerning the fit between data and tree topology: How likely are specific alternative tree topologies? What is the difference in support for distinct clades? Is the substitution model adequate? Can a clade support be explained by a bias in nucleotide substitution or in rate differences alone?

The first set of *a priori *questions is neglected in current literature. *A priori *analysis of data quality is a little explored field, and there exist few tools that are independent of tree construction. The most promising approach is to examine bipartitions (splits) that are present in a DNA-alignment, to compare their support by nucleotide patterns, and to check the compatibility of these patterns. This exploratory examination of an alignment does not need tree topologies and models. The rationale requires two assumptions: (a) If the alignment contains conserved apomorphies supporting real monophyletic groups, then these patterns should be mutually compatible. Compatibility means here that different species groups supported by patterns of nucleotides should fit to a single tree (or to a Venn diagram without intersections). Note that it is not required to infer a tree. It is sufficient to test if supported groups of species or sequences are mutually compatible. (b) The alignment is informative, if compatible signal-like patterns are based on more conserved sequence positions than contradicting (mutually incompatible) patterns. In other words, the signal should be discernible from the background noise of the data.

The first convincing tool that could be used to visualize split support present in DNA- alignments was *spectral analysis *based on Hadamard conjugation [52,53]. A nice application of the method was the study of pinniped phylogeny [13] based on mtDNA sequences. In this publication it could be shown that in an informative data set monophyletic groups show a support that is always much better than that of further incompatible splits present in the data set. This type of spectral analysis allows a correction of distances between clades using substitution models. Lento et al. [13] showed that using substitution models filters out a large part of incompatible signal. Of course, the effect depends on the model selected. However, until now this convincing visualization of effects of models has not found a broader application. One problem is that computing time grows exponentially with the number of sequences because Hadamard conjugation considers the complete split space of an alignment. Therefore computer programs like SPECTRUM [54] or Spectronet [55] can not be used on single work stations for more than 20 to 30 sequences. This is why we developed a simpler method that searches only for those splits that are represented in the data. The algorithm briefly explained below is implemented in SAMS, a new computer program developed by C. Mayer.

We present here a comparison of published phylogenies of crustacean taxa with those signal-like patterns that can be found in the original alignments used for those publications. These examples clearly show that visualization of alignment patterns tells more about the structure of the data at hand than the popular tree constructing methods.

## Results

### Strong differences in clade support visualized with split support spectra and phylogenetic networks

**Figure 1:**
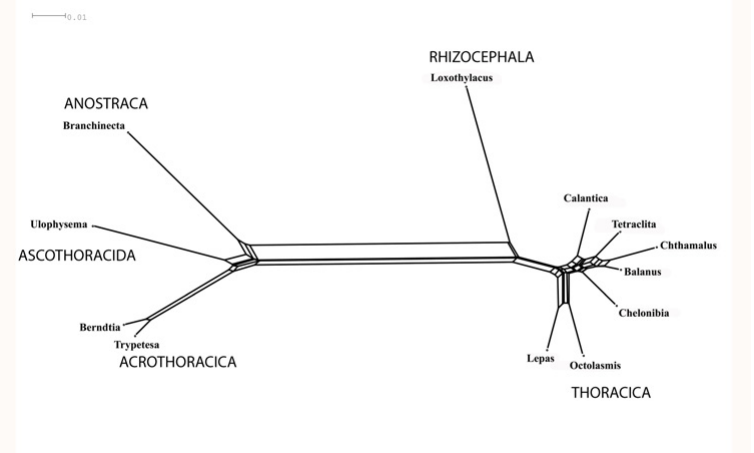
**Neighbournet network visualizing the structure of an 18SrDNA alignment with sequences of Cirripedia**. Note that with the exception of a small subnet to the right (*Calantica *to *Chelonibia*) the graph has a tree-like structure. This means that there is more signal-like information than contradicting evidence (original alignment from [55] ; outgroup: *Branchinecta*).

**Figure 2:**
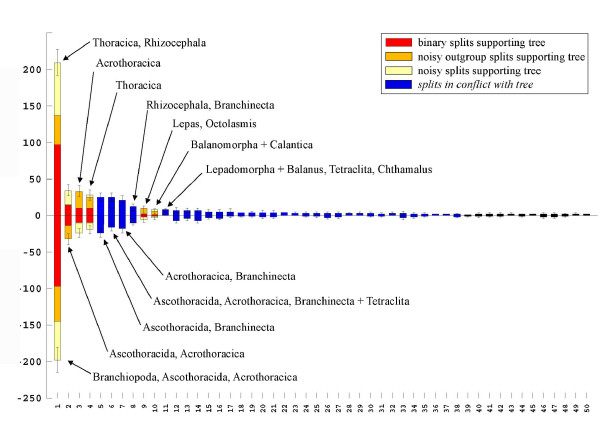
**Split support spectrum for the data used in Fig. 1**. Each column represents the number of sequence positions (indicated by the height of the column) that provide support for a given split, showing how many positions have conserved character states for each partition of a split (above and below the horizontal axis). Splits are sorted according to column height. Blue columns represent splits that are not compatible with a binary topology constructed with the strongest and further compatible splits. The four strongest splits at the left of the spectrum are the same seen in Fig 1. The right tail of the spectrum consists of random combinations of taxa.

Phylogenetic trees do not show how strong the difference in clade support really is in the raw data. We use for the following example one of the first convincing molecular analyses of crustacean phylogeny: the phylogeny of Cirripedia (Crustacea) based on 18S rDNA published by [56]. The published parsimony tree shows a long branch separating basal taxa (Ascothoracida, Acrothoracica) from the remaining Cirripedia. The same phenomenon is also seen in the phylogenetic network calculated from the original alignment (Fig. 1). Searching the alignment, one finds 245 conserved sequence positions that support the strongest split, many of these with a single character state in the ingroup. For comparison: in this data set the sessile barnacles are only supported by 3 conserved positions. Drastic differences in split support and conflicting evidence for different splits within Thoracica are also seen in the split support spectrum (Fig. 2), the differences are similar to those seen in the network.

**Figure 3:**
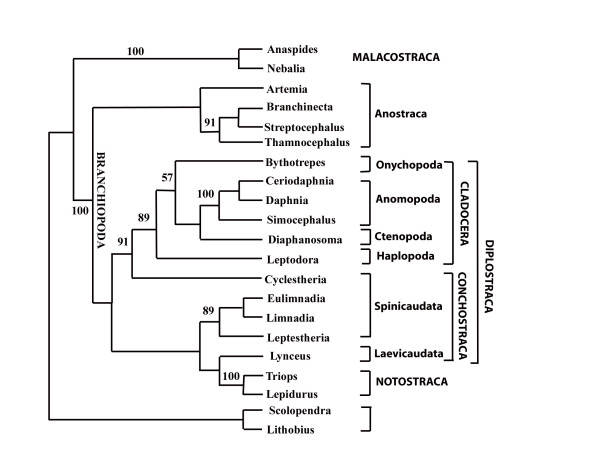
**Original tree topology estimated for 18SrDNA sequences of Branchiopoda and outgroups (Malacostraca, Myriapoda) by Spears and Abele [56]**. Compare with differences in edge lengths seen in Fig. 4.

**Figure 4:**
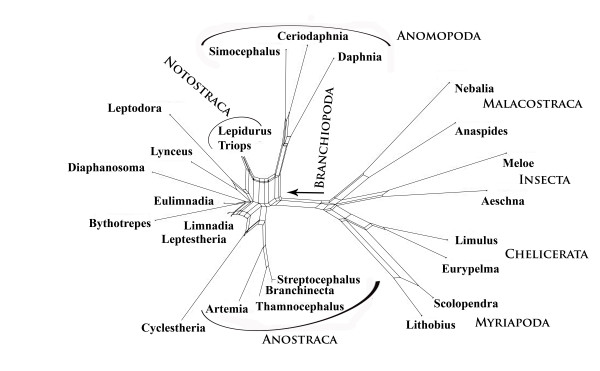
**Neighbournet graph of the data used in Fig. 3**. In this graph a split is defined by asset of parallel edges. Edge length is proportional to the weight of the associated split [30]. The strongest split separates the outgroups from Branchiopoda, and within Branchiopoda the best splits support Anomopoda, Cladocera and Anostraca. For part of the data (small subnet to the left) there is little signal. These differences in clade support are not seen in Fig. 3.

A second example concerns the phylogeny of Branchiopoda, also inferred from 18SrDNA sequences [57]. The published tree topology is shown in Fig. 3. Clades with high parsimony bootstrap consensus support are Malacostraca, Branchiopoda, Anostraca, Cladocera, Anomopoda, Notostraca. *Cyclestheria *seems to be misplaced. Usually, *Cyclestheria *is classified as genus of Spinicaudata, in the tree it appears as sistertaxon to Cladocera. The authors argue that there are also morphological characters indicating that *Cyclestheria *might not be a spinicaudatan genus. Another feature in the most parsimonious tree which is not compatible with morphology is that Notostraca are nested within Conchostraca. However, the boostrapped 50% majority rule consensus topology does not recover this grouping. Fig. 4 is a phylogenetic network based on the original alignment [57]. Obviously, for the crustacean taxa a number of conserved sequence positions contain distinct phylogenetic signals in favour of the groups Myriapoda, Chelicerata, Insecta, Malacostraca, Branchiopoda, Anostraca, Anomopoda. The Notostraca split is already weaker than the other ones. Other bifurcations of the maximum parsimony topology in the original publication are contradicted by conflicting patterns and placement of several taxa is not plausible when compared with morphological data. There are no edges distinctly better than conflicting signals that allow a safe placement of Notostraca, Spinicaudata, *Lynceus*, or *Cyclestheria*. This observation is in accordance with the observed collapse of corresponding nodes in the bootstrap consensus topology of Spears and Abele [57]. If gap sites and distant outgroups are excluded (not shown), the network is more tree-like and more similar to the current classification of Branchiopoda, with monophyletic Cladocera and Anostraca at the base of the branchiopod clade.

At this point one must remember that the phylogenetic network is not a phylogeny. In comparison with tree graphs the network (Fig. 4) clearly shows the relative differences in clade-supporting patterns and also effects of parts of the data that do not have a tree-like structure.

**Figure 5:**
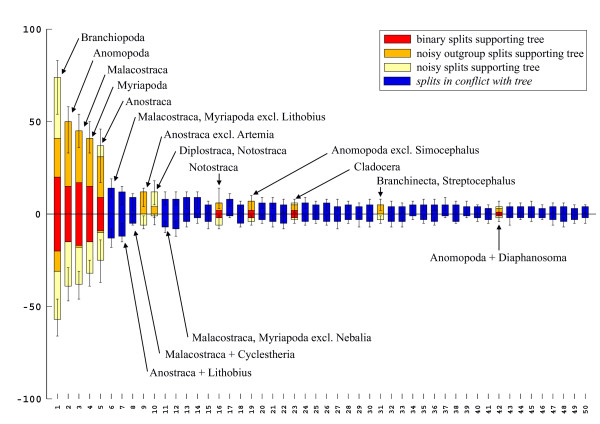
**Split support spectrum for the same data as in Figs 3 and 4 (branchiopod 18SrDNA)**. Five splits at the left part of the spectrum are clearly better than the rest. These are the same as the best splits of Fig. 4.

The split support spectrum (Fig. 5) provides information similar to the phylogenetic network. In addition, it shows a ranking order of support quality and it shows splits that are excluded in the phylogenetic network since not all splits can be drawn in a planar graph. It is clear from the spectrum that there are only 5 splits that are distinctly stronger than the first incompatible one, a product of chance similarities. Remaining splits, even those that are compatible with the tree and which make sense morphologically (e.g. for the clade {Cladocera, rest}) have no conserved support better than the background noise.

### Class I long-branch effects: the symplesiomorphy trap

A symplesiomorphy is a homology. However, it is an old conserved character state that does not substantiate monophyly of a clade [37].

In the above-mentioned publication on cirripedes [56] a basal clade was postulated which contradicts morphological data: the sistergroup relationship between Ascothoracida (represented by *Ulophysema*) and Acrothoracica (represented by *Berndtia *and *Trypetesa*, a split also seen in Fig. 1). Using maximum likelihood methods and adding more outgroup sequences, Pérez-Losada *et al*. [58] could show that this monophylum disappears. They point out that the additional outgroup sequences enable the recovery of the correct tree even with the maximum parsimony optimality criterion.

**Figure 6:**
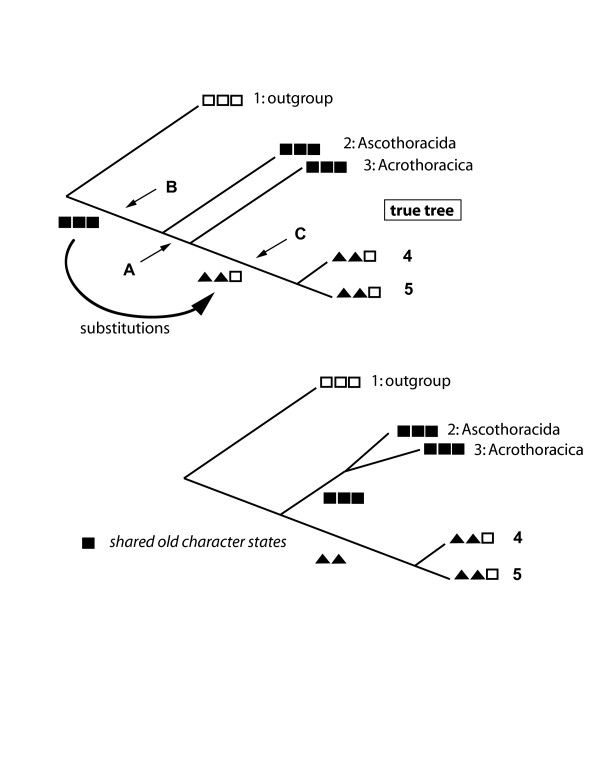
**Scheme illustrating the effect of dominating symplesiomorphies (*class I *long-branch effect)**. If old character states are substituted along the lineage leading to species 4 and 5, the clade Ascothoracida and Acrothoracica is supported by false apomorphies (in reality plesiomorphies, black squares). This effect occurred in the study of cirriped phylogeny ([55], see also Figs 1 and 2). Short inner branches (arrow A), substitutions and reversals along long branches (arrow C) increase the probability of obtaining the false tree.

These statements do not really explain the mechanism that produces the wrong topology. The problem lies in the raw data of the original alignment and is independent of the tree constructing method. It has been shown previously that the nucleotide pattern supporting the clade Ascothoracida + Acrothoracica also shares some character states with the single outgroup sequence in this data set, indicating that the supporting characters for the clade Ascothoracida + Acrothoracica are plesiomorphic [59]. This means that the supporting characters are homologous, however, they are old and did not evolve in the stem-lineage of this clade. Fig. 6 illustrates the effect: old shared similarities are substituted on the long branch and conserved in basal clades. They have the effect of synapomorphies.

This certainly is a long-branch effect, however, it is not based on accumulation of analogies, but on substitution of synapomorphies. Plesiomorphies will be retained in slowly evolving taxa and they can also be apparent plesiomorphies that evolved by "back mutations". Signal substitution increases the ratio of plesiomorphies to conserved apomorphies. This ratio decides whether the basal paraphyletic group appears as false monophylum or not. We call this the class I long-branch effect. If in Fig. 6 there would have been new characters on the short inner branch (arrow A) conserved in following taxa, or if there were no black squares substituted to other characters, the correct phylogeny could be inferred. Note that in this case the branch separating Ascothoracida and Acrothoracica is short. Therefore, the number of character states that could support the correct monophylum (in Fig. 6 {(Acrothoracica,(clades 4 and 5)} is too small.

There is a cure for this effect if there exist species that are closer to the paraphyletic group than those used for the first analysis. A species added at point B in Fig. 6 sharing character states with the outgroup would reduce the number of characters unique to the paraphylum. Adding species at point C can also help if these species conserve some of the older character states (black squares in Fig. 6).

### Class II long-branch effects: erosion of phylogenetic signal

Class I effects are based on the conservation of old phylogenetic signal in form of plesiomorphies. Branches are attracted not due to accumulation of homoplasies but by old homologies. The evolutionary mechanism can be absence of new character states for younger clades in the studied genes or subsequent substitution of synapomorphies (saturation effects on long branches). Class II effects are similar, but they require substitution of phylogenetic signal with the effect that a clade shares only character states with distantly related taxa. The resulting false group is not a paraphylum. This phenomenon has also been coined "long branch repulsion" [48], however, this term does not explain the mechanism.

Cases of signal erosion (class II effects) are difficult to detect. A conflict between morphology and molecular data in combination with the occurrence of long branches should be alarming. An example is the case of cladoceran phylogeny studied by Omilian and Taylor [60]. The data set consists of nearly complete 28S rDNA sequences of daphniids. Both maximum parsimony and maximum likelihood recovered a tree lacking a clade that is robustly supported by morphological data and also by alignments of other sequences (16SrDNA and HSP90). The clade should have been composed of *Daphnia dentifera*, *Daphnia laevis *and *Daphnia dubia*. *D. laevis *and *D. dentifera *are morphologically indistinguishable, but nevertheless are not included in the same clade in the LSU topology. Instead, the species *D. laevis *and *D. dubia *are placed at the end of a long branch, and both sequences group with *D. occidentalis*, which also has a long branch. Omilian and Taylor [60] attribute this artefact to a long-branch phenomenon. They assume that accelerated evolution of the LSU gene in the *laevis *lineage causes the observed effect. Unfortunately, no other closely related species is known that could help to break the long branches. Evidence for the existence of a systematic error comes from the already mentioned morphology and from other genes.

**Figure 7:**
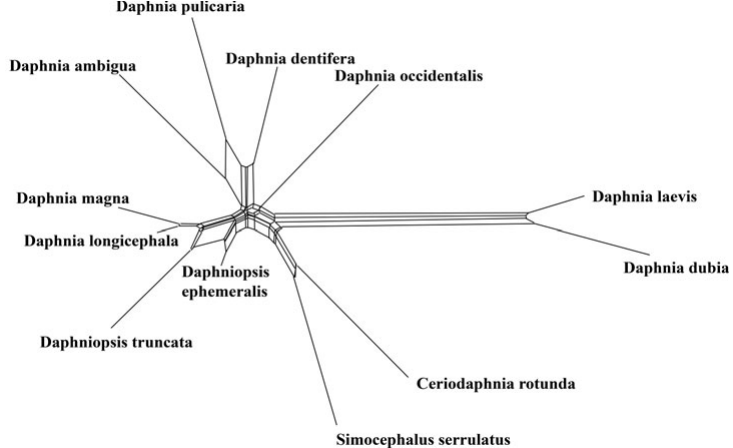
**Neighbournet graph of an 28SrDNA alignment with sequences of daphniid crustaceans**. The two species at the left of the graph clearly evolved faster than the remaining ones. The sister species (*D. dentifera*) of the fast group appears at a different place of the network. The longest branch slipped down the tree towards the outgroups (*Ceriodaphnia, Simocephalus*) (data from Omilian and Taylor [59]).

**Figure 8:**
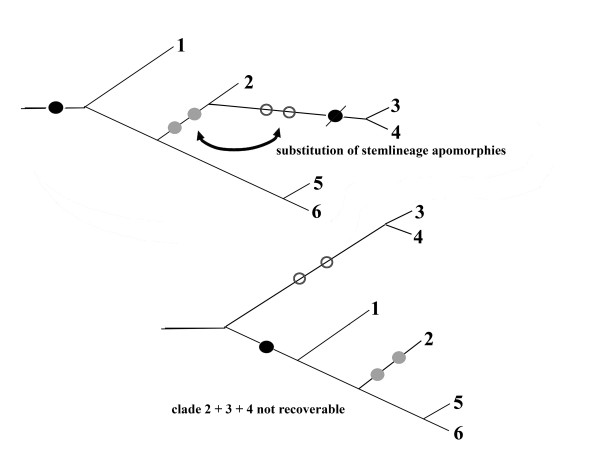
**Scheme explaining the *class II *long-branch effect**. Substitutions destroy synapomorphies on the long branch ("signal erosion") with the result that the branch slips down to the base of the tree (as in Fig. 7).

The phylogenetic network of this data set confirms the assumptions of Omilian and Taylor [60]. In Fig. 7 there are several long edges, the most conspicuous ones leading to {*Daphnia laevis*, *Daphnia dubia*} and to *D. occidentalis*. The morphologically similar species *D. dentifera *and {*D. laevis, D. dubia*} appear in different species clusters. However, these false groupings can not be attributed to shared homoplasies because removal of *D. occidentalis *does not change the situation (not shown), *D. dentifera *still retains its place at distance from its assumedly closest relatives. This indicates that synapomorphies originally shared by {*D. dentifera, D. laevis, D. dubia*} do not exist any more. The best explanation is that multiple substitutions occurred along the lineage leading to {*D. laevis, D. dubia*}, which is the longest inner edge of the topology, with the result that the correct placement of these species can not be recovered. The longest branch slips down the tree towards the outgroup taxa (*Simocephalus*, *Ceriodaphnia*). This explanation is illustrated in Fig. 8. If synapomorphies are substituted on a long branch, a monophylum can be irrecoverable and the long branch slips down to a wrong place.

The best remedy for data sets showing class II effects is the use of genes with lower substitution rates. In the case of daphniids it seems that 16SrDNA and HSP90 conserve more signal than the 28SrDNA data set.

### Class III long-branch effects: misleading and invisible attraction due to non-homologous similarities (parallel substitutions)

**Figure 9:**
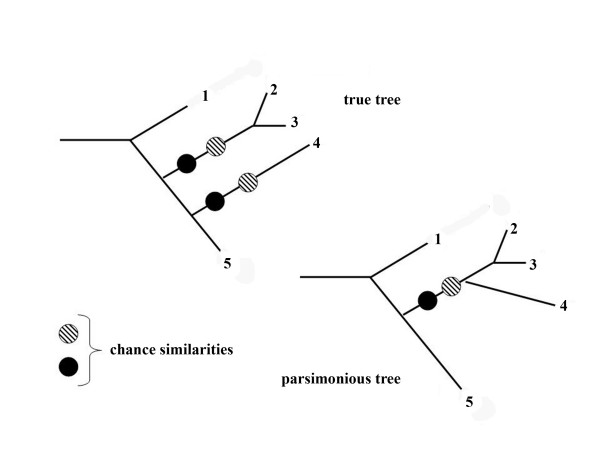
**Scheme explaining the *class III *long-branch effect**. A false clades is supported by chance similarities that evolved independently on long branches.

If identical character states evolve independently on different branches in greater number, these branches can cluster to form nonsense clades supported only by chance similarities. This is the long-branch effect that was first noted by Felsenstein [61], who found that parsimony methods are more sensitive to branch attraction than maximum-likelihood methods. The same phenomenon is well known in phylogenetic systematics when convergent morphological characters are used (e.g. the famous case of neotropical vultures that are not related to old-world Accipitridae: [62]). The basic cause for class III effects is that homoplasies can outnumber apomorphies. The mechanism is illustrated in Fig. 9.

**Figure 10:**
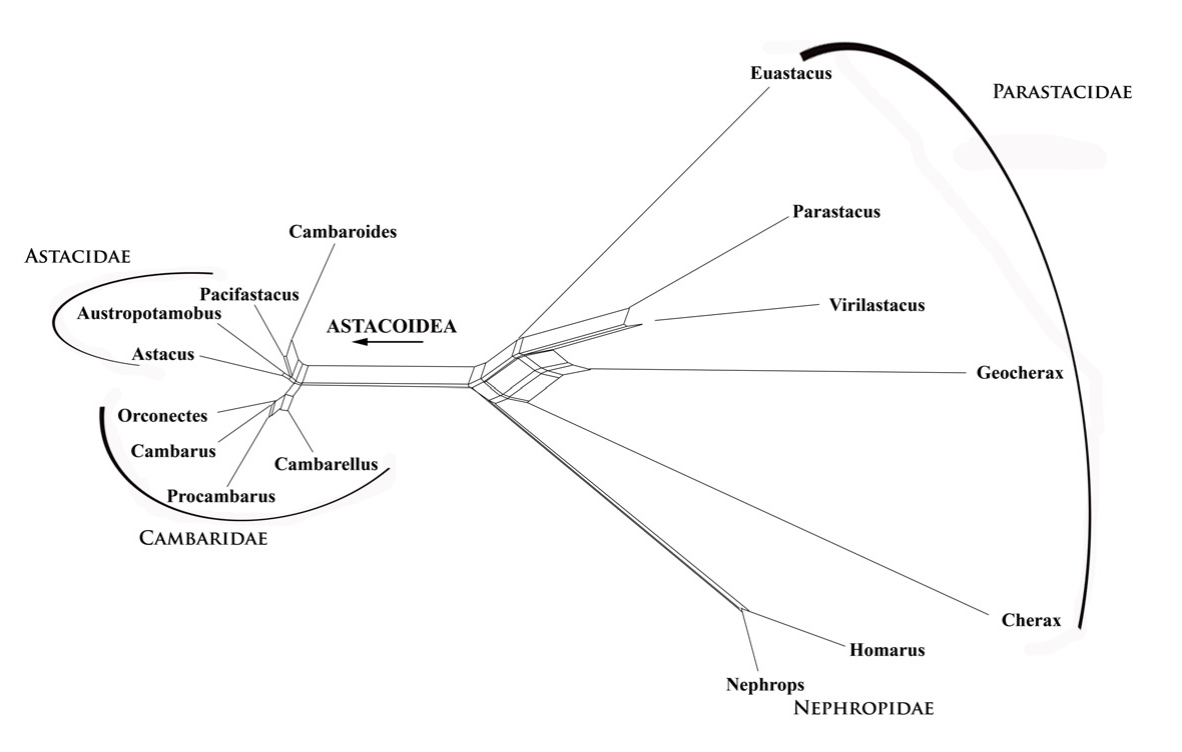
**Neighbournet graph of crayfish species based on three ribosomal genes**. Nephropidae are the outgroup. Note that some terminal branches are long (*Cherax*, *Geocherax*) (data from Crandall et al. [62]).

A case where a larger number of mutually incompatible splits exist which are invisible in the published tree topology is the study of freshwater crayfish by Crandall et al. [63] based on 16S, 18S and 28S sequences. The phylogenetic network for the original alignment (Fig. 10) shows facts not recognized in the original publication. The split between the freshwater clades (Cambaridae, Astacidae, Parastacidae) and the Nephropidae is most prominent in this alignment. Very distinct are also {(Cambaridae, Astacidae), rest)} and {(*Virilastacus*, *Parastacus*), rest}. The other clades of the original paper have a very weak support. *Cambaroides*, traditionally classified as member of Cambaridae, shares unique character states with *Pacifastacus*.

**Figure 11:**
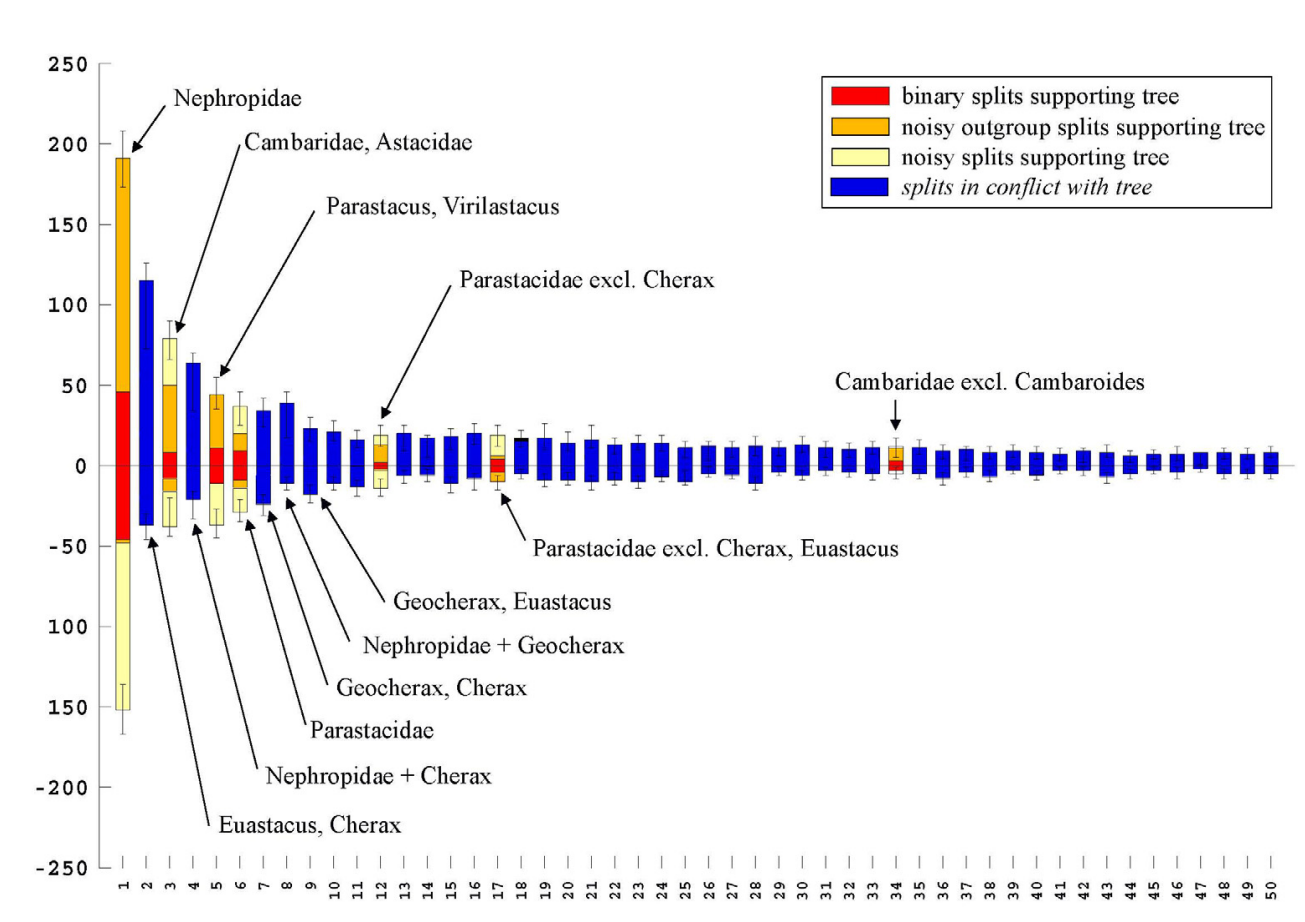
**Split support spectrum for data of Fig. 10**. There are several mutually incompatible splits containing combinations with *Cherax *and/or *Geocherax*. This is clearly a *class III *– effect.

The three most prominent splits found with SplitsTree (Fig. 10) are also the strongest mutually compatible ones in the corresponding split support spectrum (Fig. 11). However, it is clear that splits no. 2, 4, and 7 are mutually incompatible, each caused by attraction of *Cherax *to other taxa. Splits no. 7, 8 and 9 are combinations of *Geocherax *and other taxa. *Geocherax *and *Cherax *sequences form also the longest terminal branches in Fig. 10. Since these sequences are included in several clusters of species with prominent split support their placement in the published tree can be the result of class III long-branch attraction (apparent monophyly based on chance similarities). A critical clade is the Parastacidae, which seem to be more diverse and have longer branches than Astacidae and Cambaridae, implying more conflicting character states.

**Figure 12:**
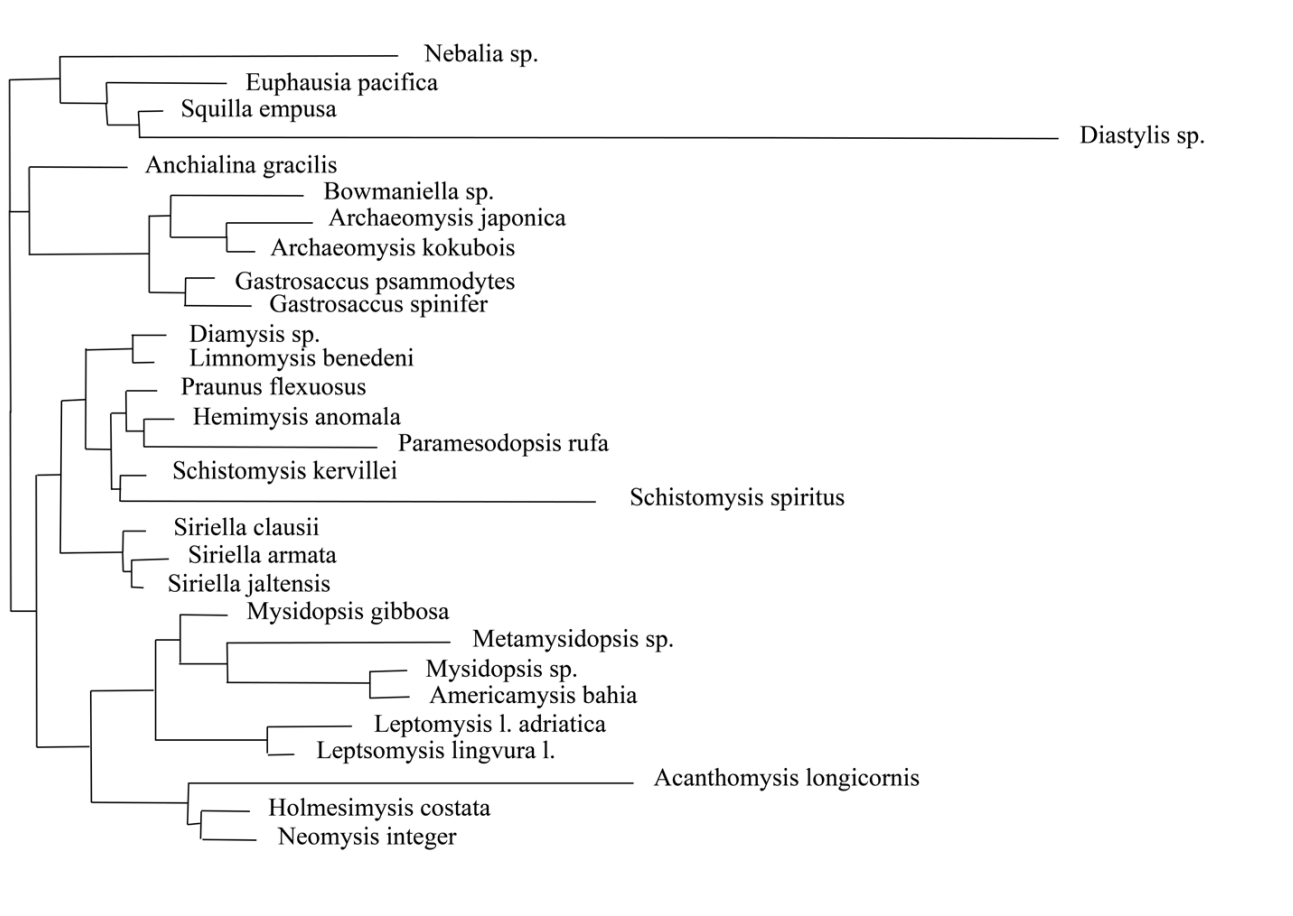
**Maximum likelihood topology for am 18SrDNA alignment of mysid crustacean sequences**. Note that three terminal branches are conspicuously long (data from Remerie et al. [63]).

**Figure 13:**
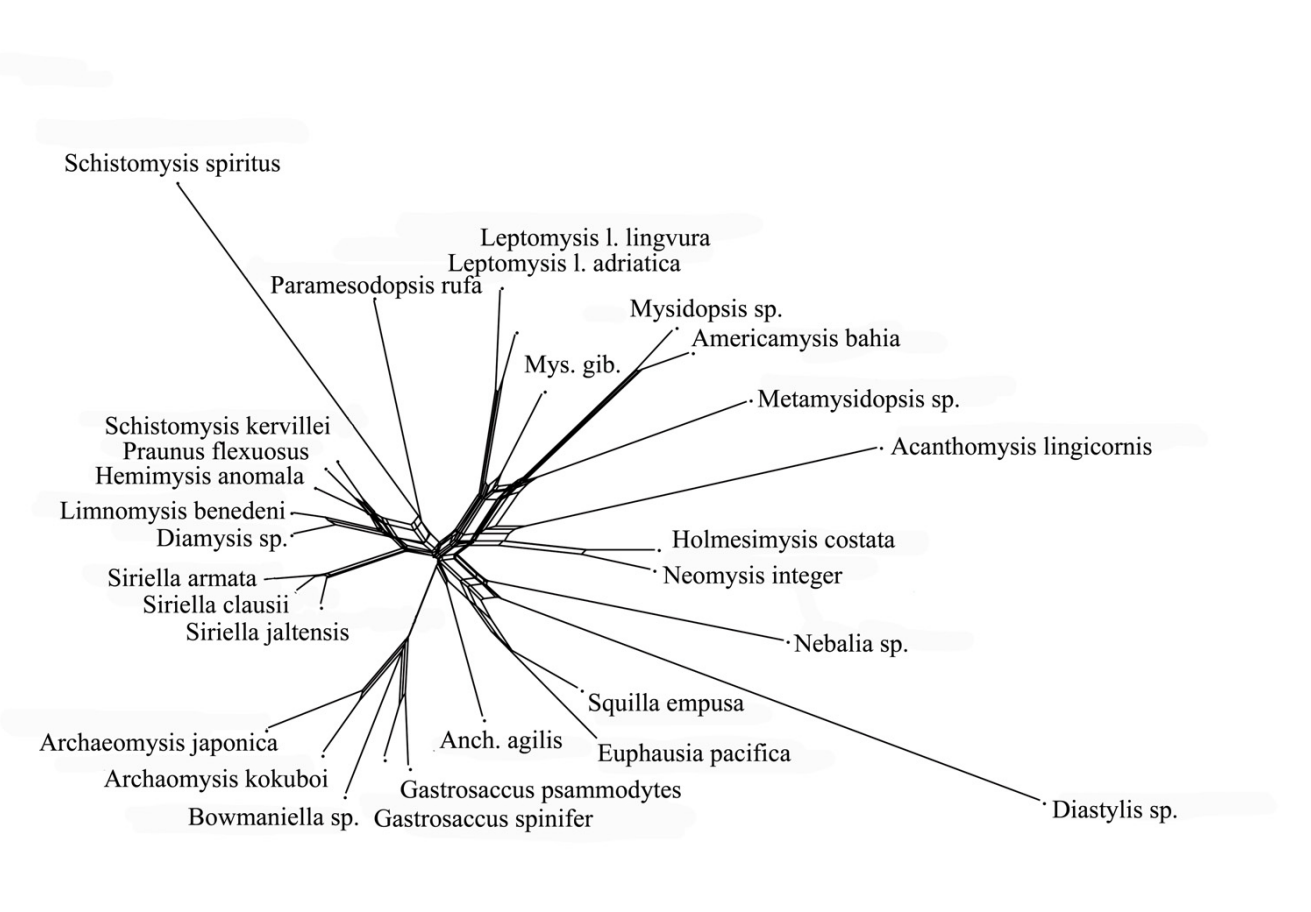
**Neighbournet graph for the same data as in Fig. 12**. Compare with Fig. 14.

**Figure 14:**
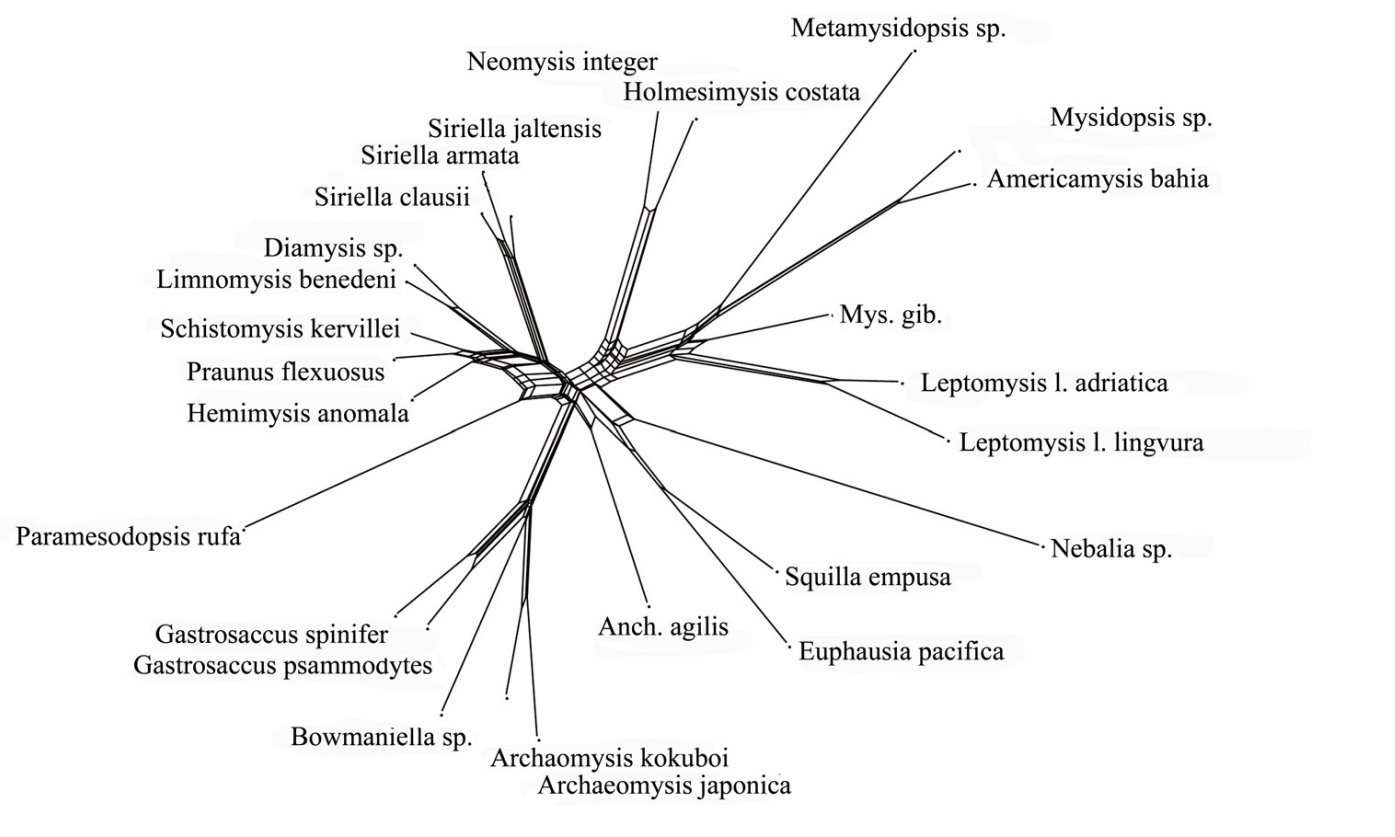
**Neighbournet graph for the same data as in Fig. 12 and 13 after exclusion of the most prominent three long-branch species**. There are fewer contradicting signals, but few changes for the major splits. This indicates that in this case the long branches have little influence on the overall topology.

**Figure 15:**
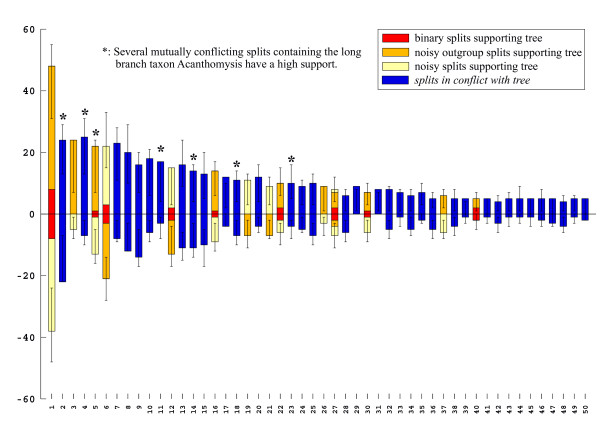
**Split support spectrum for data as in Figs 12 and 13, i.e. including long-branch sequences**. In comparison with the spectra shown before, incompatible splits (blue columns) are interspersed among the compatible ones. There are many mutually incompatible splits in combination with the same long-branch species (*class III *effect).

**Figure 16:**
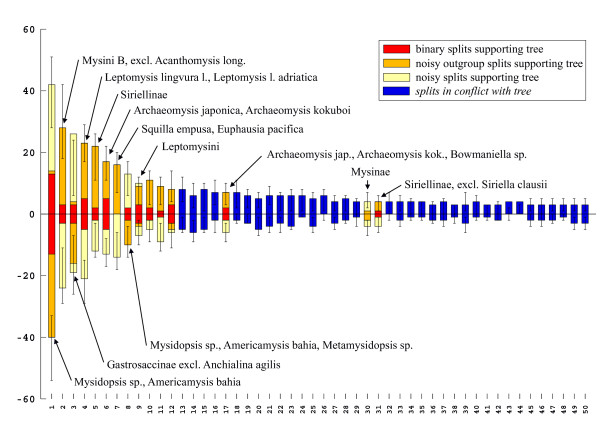
**Split support spectrum for data as in Fig. 15 after exclusion of long-branch taxa**. Note that the left part of the spectrum consists of prominent and mutually compatible splits. The alignment is informative for the corresponding clades.

Split spectra clearly improve after the removal of long branches. The difference between alignments with and without long branch taxa increases confidence in the quality of the smaller data set. An example is the 18S rDNA alignment of Remerie et al [64] used to study the phylogeny of Mysidae (Crustacea, Peracarida), where the placement of the longest branches should not be accepted without further background information. Fig. 12 shows a maximum likelihood tree obtained with a GTR + G + I model. The topology is identical with that of the original publication, however, the latter did not show branch lengths. It is obvious that there are three very long branches (*Diastylis sp., Schistomysis spiritus, Acanthomysis longicornis*) which may distort the true phylogeny. Fig. 13 is the corresponding phylogenetic network, while Fig. 14 shows the result after deletion of long branches. The well supported clades are the same as in the published topology, however, the basal branching patterns are only weakly supported. Interestingly, deletion of the three longest branches has in this case little effect on the network, but a strong effect on the spectrum. The split support spectrum of the complete data set clearly demonstrates the existence of *class III *effects caused by the long branches: Fig. 15 is a spectrum for the original data set, Fig. 16 shows how noise decreases after deletion of three long branch sequences. In Fig. 15 asterisks mark some splits that are mutually incompatible and include at least one of the long branch sequences. In Fig. 16 these splits disappear among the best 50 splits. Clearly, the reduced data set is less noisy and should be more reliable. Whether the accumulation of chance similarities influences the topology of inferred trees must be tested empirically.

### A deep phylogeny dominated by long branches and conflict

One of the most debated phylogenetic relationships is that of nematods with arthropods, which often appears in molecular phylogenies [65-74] but is contradicted by other molecular data and by morphology [30,75-86]. We will not contribute new data to this discussion but point out that many of the published data sets are not convincing. We use as an example Mallatt et al. [3].

**Figure 17:**
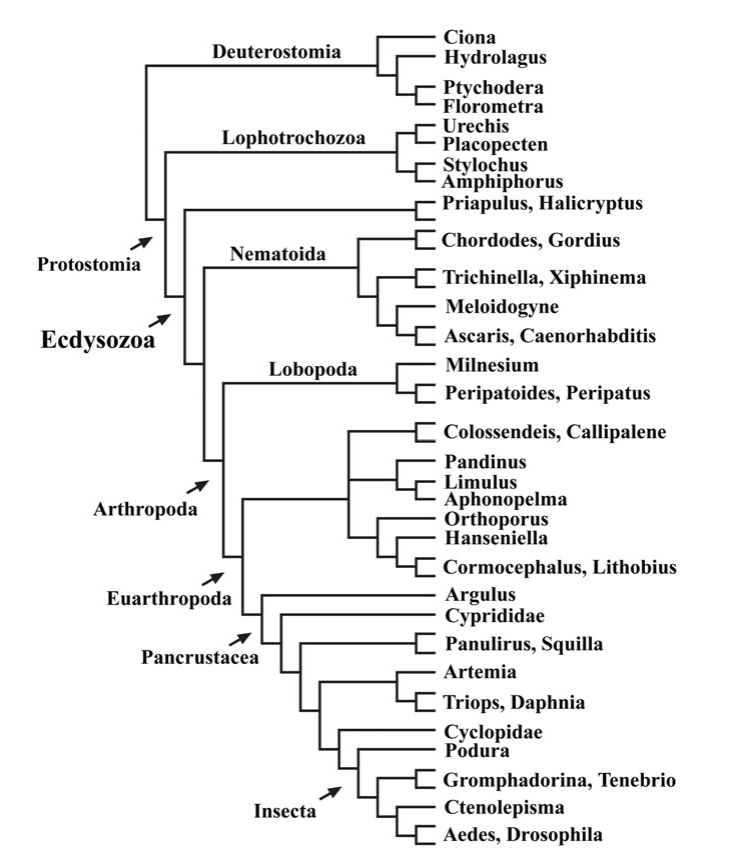
**Topology from a study of metazoan phylogeny based on two ribosomal genes [3]**. Compare with Figs 18 and 19.

**Figure 18:**
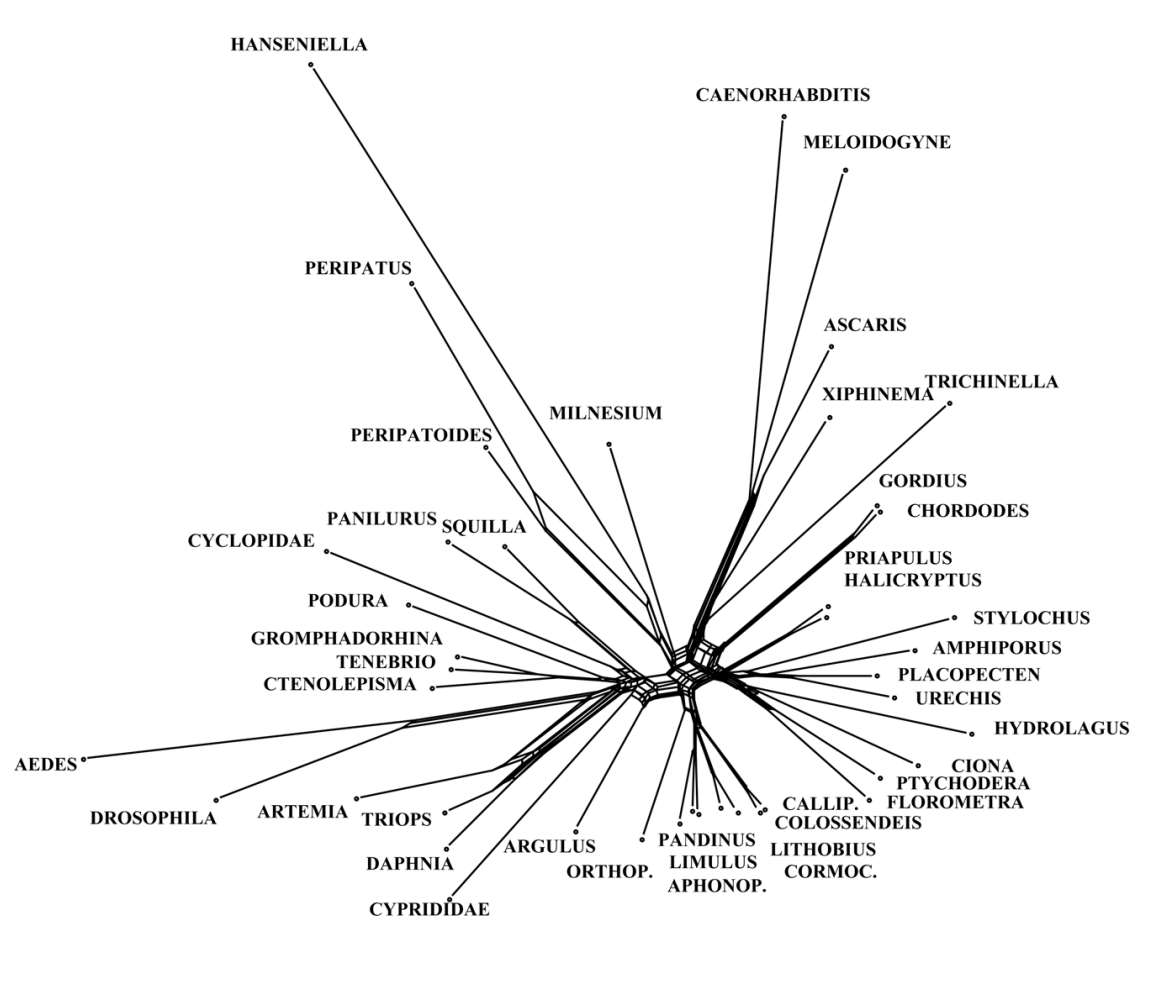
**Neighbournet graph for the same data as in Fig. 18**. The graph is dominated by many long terminal branches and many contradicting edges.

**Figure 19:**
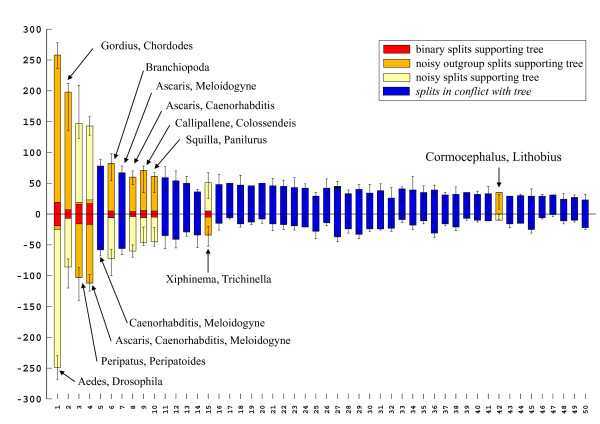
**Split support spectrum for data as in Fig. 18 (excluding the long-branch taxon *Hanseniella*)**. The most prominent splits are not interesting for the question asked (relationship of major metazoan groups). The data set is not of high quality.

A 50% majority rule consensus topology estimated with Bayesian inference from an alignment with combined 18S and 28S rRNA sequences as published by Mallatt et al. [3] is shown in Fig. 17. Many clades in this topology have high support values and suggest that this is a reliable analysis. However, the neighbournet graph (Fig. 18) reveals that this alignment is very problematic. The network contains many long terminal and few distinct internal branches. Stemminess (relative length of internal branches) increases when the longest branches are deleted (not shown), the best support in the interior of the network is that for Pancrustacea. The corresponding split support spectrum (Fig. 19) also reflects these problems: The four strongest splits are those that are of little interest (Diptera, Nematomorpha, Onychophora, Nematoda). None of the deeper nodes is found among the 50 best splits.

That the data of Mallatt et al. [3] are not reliable is also indicated by strong contradictions with morphological data. For example, the published tree has a "mixed" clade composed of insects and copepods (bootstrap support: 99). Until now not a single morphological character is known that allows to postulate such a phylogeny. Another clade has the structure {scorpion, {*Limulus*, spider}} (bootstrap support: 100). This would imply polyphyletic Arachnida. Of course, it is possible to get a binary tree from weak data. Such a tree shows the best of all mutually compatible splits, and bootstrap or Bayesian support can be high if the real phylogenetic signal eroded along long branches. However, it can not be recommended to trust in these results because nonsense splits can also be mutually compatible in an optimal tree.

### Phylogenetic signal drowned in noise

**Figure 20:**
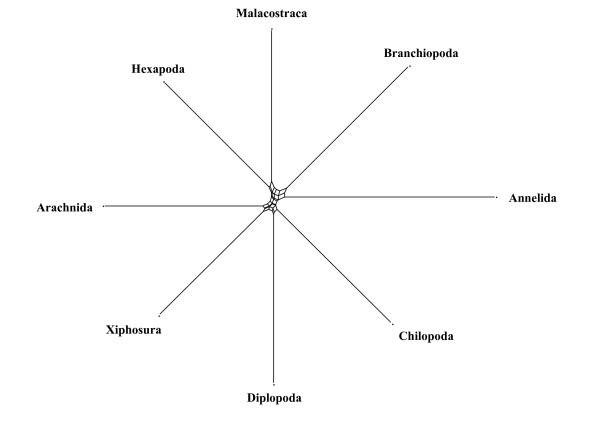
**A „Cambrian explosion" neighbornet**. Even though the alignment was long (more than 20,000 bp) the data set is of no value. Informative positions contain many autapomorphies for terminal taxa. There are few group-supporting signals, and these are contradicting each other. Data published by Pisani et al. [84] (see also Fig. 21).

**Figure 21:**
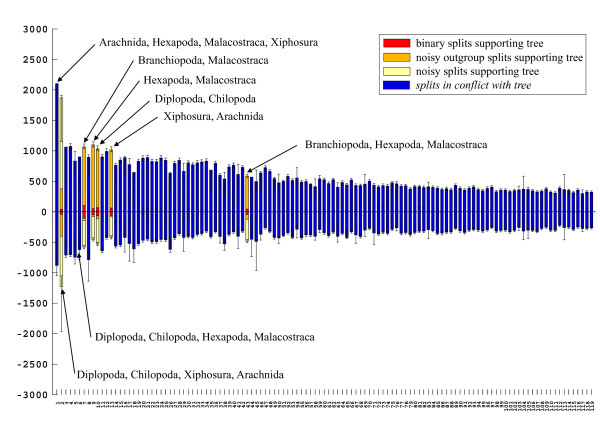
**Split support spectrum for data as in Fig. 21**. All possible bipartitions of the set of taxa are supported. It is not possible to distinguish between signal and noise. Compare with Figs 2 and 16, where this difference is obvious.

Of the examples presented herein, the worst is the data set published by Pisani et al. [87], a contribution dedicated to arthropod phylogeny. The authors concatenated nine nuclear and fifteen mitochondrial genes, however, sequences are hybrids composed of fragments from several species belonging to the same clade, wherefore a sequence is named e.g. "Branchiopoda" and not "*Artemia salina*". There are only eight hybrid sequences. The authors stress that they used a very long alignment (21,313 bp), that inner nodes got high support values, and that support for a close relationship between myriapods and chelicerates is consistent. The number of sequence positions is larger than the 10.000 proposed by Dopazo et al. [79] as necessary to obtain a correct tree, but nevertheless the data set is not convincing:

The exploratory analysis of this data set is disappointing. The phylogenetic network shows a Cambrian explosion without internal resolution (Fig. 20). Fig. 21 is the spectrum for this alignment. In this case we show the complete spectrum. It contains all possible 127 splits obtainable from combination of 8 taxa and there is with two exceptions no distinct increase in signal at the left part of the spectrum. The exception are the first two splits, which unfortunately are mutually incompatible. This means that it is not possible to discern here with conserved alignment positions any phylogenetic signal that is better than the background noise. The high bootstrap support should not surprise. It is known that in large alignments bootstrap replicates will all be very similar [30].

## Discussion

We do not intend to discuss here the phylogeny of Metazoa or of Crustacea or all methods proposed in published literature to improve phylogeny inference. It is the goal of this contribution to show that data used for published trees differ extremely in signal to noise ratio despite comparable good node supports. These differences can be observed only when using tree-independent methods of visualization of patterns actually present in alignments.

In our analyses, noise is detected when splits are mutually incompatible. Excluding cases of horizontal gene transfer (which are very rare among animals), the only explanation for incompatibility is that shared character states are not homologous at least in one of two mutually contradicting splits. To demonstrate effects of noise we did not use simulations, but analyzed real data which show unpredictable patterns. These patterns are real, each position and nucleotide contributing to a pattern can be identified, and nothing is transformed by model assumptions.

For example, the alignment of cirriped rDNA has few splits with very strong support in comparison with the right tail of the spectrum (Fig. 2). The support for these outstanding splits certainly can not be explained with accumulation of chance similarities. In contrast, the data set of Pisani et al. [87] has no mutually compatible splits with a distinctly better support than the multitude of incompatible splits representing all possible taxon combinations (Fig. 21). There is no evidence for the presence of compatible signal-like patterns that can not be explained with chance similarities alone.

It has often been shown that some of the problems caused by noise can be overcome with adequate substitution models and also that inadequate models can converge on the wrong tree [e.g. [5,88-99]]. There exists a huge literature on modelling of sequence evolution and on adapting model parameters to empirical data. However, two fundamental problems have not been solved:

(a) As the history of population dynamics in ancestral lineages and the real historical effects of selection and of population size on site and rate variability usually remain for ever unknown, substitution models will always be nothing but averaged approximations. There exists no test for how close to the historical reality a model is.

(b) There is no test to examine whether available raw data are good enough to find the correct model parameters. Models of sequence evolution can be adapted to patterns present in a real data set. However, it has never been asked how to test if the information content of an alignment is sufficient to find a realistic model.

Simulation studies have shown that even if the real model parameters are known, a tree found by a maximum likelihood method with carefully adapted model parameters may not be optimal or may not be the only optimal topology [e.g. [100]]. In the end, the probability that trees are based on historical signal is correlated with the amount of information conserved in the raw data, no matter what method of tree inference is used.

Grant and Kluge [101] evaluated in their comprehensive review a large number of methods used for data exploration. In contrast to split support spectra, none of these methods is tree-independent. The authors distinguish between *sensitivity analyses *(the responsiveness of conclusions to changes or errors in parameter values and assumptions) and *quality analyses *(assessment of abilitiy of data to indicate the true phylogeny). Examples for sensitivity analyses are the bootstrap, jackknife, and other methods that use pseudoreplicates of character distribution of the same set of data, and also the Bayesian phylogenetic inference, Bremer support, and Wheeler's sensitivity analysis [102,103]. For a critique of these methods see [101].

Beside bootstrapping, popular tools frequently used by systematists were for some time the estimation of consistency and of tree length distribution under the parsimony criterion [104-108] and derived tests that measure departure from a model of randomness. An example for an elaborate detection of noise with these tools is the study of the phylogeny of Trichoptera by Kjer et al. [109]. The authors analysed the skewness of tree length distribution for the complete data set and for subsets of taxa, the accumulation of substitutions along a "highly corroborated tree", the consistency index as guide for character weighting. These approaches depend on the assumptions of the maximum parsimony method or of other optimality criteria and do not identify conflicting splits and differences in the quality of clade support.

Data quality is for us an estimation of the probability that phylognetic signal is conserved, without reference to a tree topology. Several methods have been proposed to identify quality of alignments in this sense. Grant and Kluge [101] list among this class of methods spectral analyses, RASA, and data partition methods. RASA (relative apparent synapomorphy analysis: [36]) is a method that counts the number of characters shared by two taxa in a three-taxon comparison. A test statistic is derived essentially from the rate of increase in pairwise shared character states in comparison with a null model based on randomly distributed characters (for further details see [36,110,111]). Simmons et al. [33], using hypothetical and empirical examples have shown that RASA is not effective to measure phylogenetic signal.

Spectral analysis in the sense of Hendy and Penny [52] is a tool that visualizes the treeness of data and the amount of conflict. The more tree-like the data are, the higher is the probability that shared similarities are homologies. Our SAMS method also must be classified as a quality analysis. In this case, the stronger the support of the best compatible splits is, the higher is the probability of homology for character states in corresponding supporting positions.

Strategies to improve the signal to noise ratio discussed in many publications comprise increased taxon sampling, addition of more genes, deletion of highly variable positions (e.g. third codon positions), R-Y recoding, deletion of highly variable sequence regions. The application of better models of sequence evolution is another option that does not involve manipulation of raw data. The more costly approaches are increased taxon and gene sampling. To reduce problems caused by the frequently cited long-branch effects [e.g. [61,112-117]] it is important to know whether it is more promising to collect additional species or to sequence additional genes. To decide this it is relevant to distinguish the three long-branch effects.

*Class I long-branch effects *(the symplesiomorphy trap) can be overcome with better taxon sampling as already explained above (Fig. 6). A data set may contain strong signals consisting of plesiomorphic character states and erroneously supporting monophyly of paraphyletic groups. If long internal branches are detected, one should try to find species that probably attach to these. Addition of more genes would not help because increasing the number of shared plesiomorphic homologies would only stabilize the wrong grouping. The advantages of increased taxon sampling, especially when taxa addition is not random but controlled by the investigator, have been noted earlier [e.g. [118,119]], however, an explanation of the possible mechanisms was missing. For example, Pollock et al. [120] made simulations in which phylogenetic reconstruction was affected more by taxon addition and by sequence length than by moderate variations in substitution rates. The fact that symplesiomorphies may be a problem was not discussed. Wherever addition of a taxon changes the topology, the breakup of class I effects may be the underlying mechanism.

It has been observed earlier that attraction by symplesiomorphies is a phenomenon that occurs simultaneously with long branch attraction, typically when the four-taxon case is studied [e.g. [77]]. In the latter case, the two long branches share analogies, the shorter ones share conserved character states. This is not the same as the *class I *effect defined herein: terminal taxa sharing symplesiomorphies must not necessarily evolve slowly (Fig. 6), and a single long internal branch can already cause the effect when synapomorphies erode along this branch.

*Class II effects (signal erosion) *can not be cured by addition of taxa. What is needed are slowly evolving genes that hopefully conserve old homologies. Spectra can be used to control improvement of the signal to noise ratio with increasing alignment length [121].

Class II effects are expected to be more frequent in deep phylogenies. The probability that apomorphies are substituted increases with time. This has two consequences: the certainty for the identification of homologies decreases with the age of clades, and the evolutionary rate of genes may seemingly slow down with age because the number of conserved apomorphies decreases [122,123]. As a consequence, taxa with long branches may be placed at the base of a tree because of erosion effects. Stiller and Hall [116] discovered that clustering of eukaryotic protists basally of a crown group can be explained entirely by the sequential attachment of longer branches in the absence of phylogenetic signal. Morin [124] describes long branch "attraction" as responsible for the basal placement of Diplomonadida, Microspora and Parabasalia at the base of the Eukaryota. This certainly is another case of signal erosion and not of artificial attraction.

*Class III long-branch effects (misleading attraction due to non-homologous similarities) *are caused by stochastic accumulation of non-homologous character states due to high substitution rates, old age of lineages, but also by systematic biases such as convergent shifts in nucleotide frequencies. It is well known that to strengthen the phylogenetic signal it would be useful to search for biases (e.g. comparing nucleotide frequencies in different genes for the same clades), to remove terminal long branches, to break long branches by adding taxa, and to consider alternative and concatenated genes. Of course, a long alignment is no guarantee for the recovery of the real phylogeny [e.g. [125]], since more genes will not necessarily correct biases, the effects of wrong substitution models and class I long-branch effects. Genome-wide phylogenetic analyses [e.g. [82]] are promising, however, the small number of available taxa bears a risk [77] because topologies are composed of many long branches. Another strategy that can help is to delete highly variable sequence positions to reduce the number of non-homologous similarities. The effects of noise reduction are immediately visible in split support spectra (compare e.g. Figs 15 and 16).

It has been shown that long-branch effects (usually referring to class III effects) are not only a problem occurring with maximum parsimony methods; maximum likelihood is not immune to long-branch attraction [48,59,126]. It is therefore interesting to identify possible sources of long-branch effects no matter which method of tree inference is used. In most publications no difference is made between attraction due to accumulation of analogies, the dominance of symplesiomorphies, or slipping of a branch down the tree due to signal erosion. An interesting test for putative occurrence of long-branch effects is the simulation of sequence evolution along topologies, alternatively with and without junction of long branch taxa, followed by an analysis of the artificial data to check for deviations from the true tree [117]. A problem may be that branch lengths can not be determined accurately when sequences are saturated in more variable regions (hidden long branches). If parsimony and likelihood topologies differ or if a likelihood model with equal rates gives a topology different from a tree obtained considering rate heterogeneity, then class III phenomena (accumulation of chance similarities) can be the cause.

The saturation phenomenon is part of the long branch problem. Multiple hits can destroy signal and cause class II effects when too few conserved synapomorphies remain after some time, or they cause class III effects when substitutions produce non-homologous similarities.

What are the implicit assumptions of SAMS? The calculation of split support spectra as implemented in SAMS is based on the assumption that the probability of homology for shared nucleotides is larger if (a) sequence positions are conserved and if (b) a large number of sequence positions support the same split. Conserved positions are those that evolve slowly, implying that multiple non-homologous substitutions should be rare in such sites. Taking the sum of positions as a single pattern supporting a split, then splits with larger, more complex patterns are thought to be more reliable as potential traces of phylogeny than splits with patterns consisting of few positions (criterion of character complexity as discussed in [121]). As expected, the spectra of split supporting positions always show many hundreds of incompatible splits supported by few (e.g. 2–5) positions, but only few that are backed by a large number (e.g. in Fig. 2). The latter are less prone to stochastic effects.

The criterion of character complexity used to evaluate support spectra is not the same as the congruence criterion used in cladistics because cladistic congruence [127,128] requires a tree topology and does not discern between character qualities (slow vs. fast evolving characters). The selection of positions that contain split-supporting information is a sort of differential weighting. However, in contrast to successive weighting schemes it is not intended to select characters and weights that maximize congruence on a most parsimonious tree, but we select characters that maximize the probability that a support consists of homologous nucleotide patterns in a topology-independent exploration of the alignment. Grant and Kluge ([101] p. 411) complain that "most methods of quality analysis function as data purification routines, whereby evidence is discarded or manipulated to make it conform with some notion of goodness". We confess that we also want to discard part of the characters, namely those which bear with less probability traces of phylogeny and which introduce noise in the data set (difference between Figs 15 and 16). We are convinced that this is legitimate and that the search for reliable evidence is good practice in science. However, we want to avoid circular argumentation, e.g. we do not select characters that fit to a tree.

Grant and Kluge [101] point out that explorative methods should perform empirical tests with the potential to refute a hypothesis. The spectral analysis implemented in SAMS fulfils this requirement. Empirical data are used to test either if clades proposed by previous phylogenetic analyses are represented with nucleotide patterns that are stronger than the background noise, or to test if a given data set contains distinct signal-like patterns.

The *a priori *analyses presented herein are fast tools for the assessment of data quality and we found that results are intuitively comprehensible. However, we must confess that even though the split support spectra proved to be from our point of view of heuristic value, the method is still imprecise and needs further development. The threshold of position variability that defines which positions are accepted as part of a supporting pattern is chosen arbitrarily (25% variation per column and group) and is conservative, rejecting many positions that may still fit to a pattern. Therefore, our spectra show only the contribution of slowly evolving positions. A more objective method, e.g. derived from entropy theory, or a more flexible tool that allows selection of positions according to rate differences is still missing. Also, simulations are necessary to study in greater detail effects of systematic biases.

## Conclusion

Split support spectra and network analyses are not meant to replace tree building methods. As used herein, the spectra show only distinct conserved patterns, and many clades that appear in trees are not represented among the best splits whenever they are supported by very few conserved positions. This does not mean that such clades are not real. If some groups of species are closely related, the number of synapomorphies will be small. However, spectra and split networks will show whether the complete alignment contains distinct signals or not, whether a clade is strongly contradicted, and which clades have the best support. If mutually compatible signal-like patterns that surpass the background noise are absent, any tree derived from such an alignment may contain mainly nonsense clades.

We found in the examples studied by us that usually all compatible strongest splits are also represented in trees obtained with optimality criteria. They determine the topology. The remaining clades of this topology contain only those of the weaker splits that are compatible with the strongest ones. There is one exception, where strong signals inspire trust in an incorrect phylogeny, namely when class I long-branch effects occur. Plesiomorphies are homologies and therefore real historical signal, however, they do not prove monophyly. Distinction of different types of long-branch effects as defined herein will help to decide how to improve molecular data sets.

## Methods

A split is a bipartition in a species set, which separates two groups that comprise together all species of the data set [129-132]. To find splits present in a data set and to visualize split support we used SAMS. This software written by one of us (CM) is a successor of the program PHYSID used for previous publications [34,84,133-135].  An executable file, a manual and example data blocks are attached here as additional files (files 1,2,3,4,5).  SAMS (Splits Analyses Methods, available from CM upon request) is a tool that searches for splits present in a data set. The method does not estimate distances between clades, but offers simple counts of sequence positions that fit to a bipartition. The absolute number of positions is not relevant, it is only informative within a split support spectrum (Fig. 2). The comparison of split support in spectra allows to discuss competing hypotheses. These hypotheses can refer (a) to the monophyly of a group of species or (b) to the phylogenetic information content of an alignment (e.g. "gene A is better than gene B to infer the history of taxon X"). If proposed clades are weakly supported, the conclusion is that evidence for this clade is poor or lacking in a given alignment. This does not necessarily mean that the group is not monophyletic.

The most conserved informative positions supporting a clade are binary, i.e. with two character states only. Each character state is potentially a plesiomorphy or an apomorphy for a group of a split. Since the real monophylum and its outgroup are not identified to avoid prior assumptions, it is sufficient to observe that a position clearly supports a certain split. Binary positions are rare, especially if taxon samples are large because substitutions can occur on any branch within a clade. Therefore, SAMS adds to a split-supporting pattern also noisy positions, i.e. positions with more than two character states, if a majority state within a group still can be identified. One should expect that noise has a random distribution in supporting positions. If deviations from a majority character state accumulate in a single sequence, the corresponding characters could be plesiomorphic states. This phenomenon is observed in sequences of slowly evolving or of less derived taxa. Therefore positions with putative plesiomorphies are not counted (see also [34,121]).

SAMS does not polarize characters. It can be used without knowledge about the root or the real outgroup species.

Split support is visualized similar to Lento plots [13]. Since character states are often conserved within one taxon and variable in the outgroup, SAMS counts supporting positions for each group of a split separately. Thus, for each split of a data set two numbers of supporting positions are given in the spectra, one for each group of species, shown above and below the horizontal axis (Fig. 2).

In Figs 2, 5, 11, 15, 16, 19, and 21 splits (columns) compatible with a published topology are shaded differently from incompatible ones. In addition, in each column three different types of positions are discerned: binary (only two character states), asymmetrical (one partition of the split with only one character state, the other with more than one state), and noisy positions (more than one state in each partition). For further details see [34,135]. If not stated otherwise, the spectra show only the 50 best splits. We only labelled splits of special interest, however, SAMS allows the identification of every split.

SplitsTree V.4 was used to calculate phylogenetic networks (see [30] for a review of applications). We compare the network structure based on the neighbornet algorithm [136] and applying the LogDet transformation [95,137,138]. LogDet is a distance transformation that corrects for biases in base composition. Alignments were obtained directly from the cited authors. Some case studies could not be carried out because the original alignments were not available.

## Authors' contributions

CM developed the algorithms and wrote the software SAMS and calculated the split support spectra. WW developed earlier the principle of these analyses [34,84,121,135], prepared phylogenetic analyses using published alignments, constructed the split networks, drafted the manuscript and discussed the results.

## Supplementary Material

Additional file 1 SAMS nexus block example (1). Example for SAMS commands in nexus format: Reading a data file. Excluding character positions or taxa from the analysis. Analysing base frequencies. Exporting the data set to different file formats. Click here for file

Additional file 2 SAMS nexus block example (2). Example for SAMS commands in nexus format: Reading a data file. Exluding character positions or taxa from the analysis. Computing split supporting positions. Click here for file

Additional file 3 Alignment example in nexus format. Data set from Remerie et al 2004: Phylogenetic relationships within the Mysidae (Crustacea, Peracarida, Mysida) based on nuclear 18S ribosomal RNA sequences, Mol. Phyl. Evol., 32(3), pp 770–777. Click here for file

Additional file 4 SAMS manual. Manual with description of functions and commands of the software SAMS Click here for file

Additional file 5 SAMS. Executable file for the software SAMS Click here for file
