# Supplementary material for: Visualizing differences in phylogenetic information content of alignments and distinction of three classes of long-branch effects
Source: BMC Evol Biol. 2007 Aug 28;7:147. doi: 10.1186/1471-2148-7-147 (PMC2040160; doi:10.1186/1471-2148-7-147)
Supplement: Additional file 4 — SAMS manual. Manual with description of functions and commands of the software SAMS [file 1471-2148-7-147-S4.pdf]

SAMS

(Splits analysis methods)

Version 1.4 beta

Christoph Mayer
and
Wolfgang Wägele
2005

Disclaimer:
The SAMS-program (version 1.4 beta) is a beta-test version which is still under development. The authors are not aware of bugs that would cause the program to obtain incorrect results, but they could exist. Even though the authors try to make this program as reliable as possible, it could be that parts of the program do not work as intended. Please note that in this test version the number of taxa that can be analysised is limited to 256. Please report any crashes, bugs, or problems you have with this program. This program is distributed in the hope that it will be useful, but without any warranty.
This manual is still under construction. Therefore, some features of SAMS are only briefly documented in this manual.

License:
This program is copyright protected. Results obtained with this program can be published without restrictions, provided the program and its authors are acknowledged by name. Future versions of this program are intended to be released under the "Gnu Public License" (GPL). Since the current version is a pre-release version distributed merely by request from the authors, it is not shipped with source code or the GPL.

New versions of SAMS and of this manual will be made available from the following web-page: `ttp://www.rur`-uni-bochum.de/spezzoo/cm/cm.html* .

Contents

1 Command Reference **1**

1.1 Introduction 1

1.2 Input file format 1

1.3 The SAMS-commands 2

1.3.1 The command and option syntax 2

1.3.2 Save output to a file 2

1.3.3 Unix commands within SAMS 3

! 3

cd 3

ls 3

1.3.4 The general SAMS commands 4

Assume 4

Delete 4

Exclude 4

Execute 5

Help 5

Include 5

Log 6

PrSequences 7

Restore and Undelete 7

SetGlobalParameters 8

Show 8

ShowGlobalParameters 8

Undelete 9

1.3.5 General analysis commands 9

PrBaseFrequncies 9

1.3.6 Commands in the PHYSID module of SAMS 9

PhysidBootstrap 9

PrPhysidBootstrap 11

PhysidDetSupPos 12

PhysidMultiSplitsDetSupPos 12

PrPhysidSupPos 14

PrPhysidMultiSplitsSupPos 14

SetPhysidParameters 16
ShowPhysidParameters 16

Chapter 1

Command Reference

1.1 Introduction

SAMS is a command-driven interactive analysis program for molecular data. It implements several routines which, for a given set of aligned DNA sequences, estimate the phylogenetic signal present in the data that supports or contradicts putative splits, i.e. internal branches in putative phylogenetic trees. With this information it is possible to visualize the information content of the data set and the signal to noise relationship.

Currently, two methods are available to determine support values for splits: (1) The so called PHYSID method developed first by Wägele and Röding (1998) and refined by Mayer and Wägele (2005), and (2) the Shannon-Entropy method developed by Mayer and Wägele (2005). (Method (2) is not yet available in the current release version of SAMS). Both methods have in common that they do not refer to a tree topology or a model of sequence evolution, and are therefore an ideal tool for a priori estimation of information content of data sets.

1.2 Input file format

SAMS is capable if reading data files in the NEXUS format[1] (Maddison *et al.*, 1997). It can read all relevant NEXUS-blocks including Taxa-blocks, Data-blocks, Character-blocks, Assumptions-blocks, and Tree-blocks. Blocks that are unknown to SAMS are ignored. In the same spirit as other programs that are using the NEXUS-format (such as PAUP, MacClade, and MrBayes), SAMS defines its own block, the so called SAMS-block, in which the user can write any set of SAMS-commands in order to *drive* the program.

[1]A short description of the NEXUS-format can also be found in the PAUP manual (Swofford 2003).

1.3 The SAMS-commands

The commands described in this section are specific to SAMS. They can either be passed to SAMS by typing them from the command line or by including them in a SAMS-block in a NEXUS-file. In both cases, commands and options can be abbreviated as long as they are unambiguous.

1.3.1 The command and option syntax

A general SAMS command has the following syntax:

$\texttt{command-name option-name}_1\texttt{=value}_1\ \texttt{option-name}_2\texttt{=value}_2\quad \ldots\quad ;$

where no or any number of options can be passed to the command. Each option consist of an *option name*, an "=" sign, and the value assigned to its name. There is just one exception: For options which can be set to *'yes'* it is sufficient to write merely the *option-name* instead of *option-name = 'yes'*. To give an example, we abbreviate the **append=yes** option by writing **append**.

On the command line, several commands can be entered on a single line if they are separated by colons. A missing colon after the last command will automatically be appended to the line.

If the commands are passed to SAMS in a SAMS-block in a nexus file, the usual nexus file syntax applies. In particular, the line structure of the file is irrelevant for its interpretation (commands and options can be formated freely), and each command has to be terminated by a colon.

1.3.2 Save output to a file

By default, all output commands write to the screen (stdout) and, if specified, also to a log file. In order to redirect output to a file, most output commands provide the following options:

```
[ file = <path-file-name> ]
[ append = no|yes ]
[ replace = no|yes ]
[ genfilename = no|yes ]
```

If the **file=<path-file-name>** option has been specified, the output is written to a text file instead of the screen. Here **<path-file-name>** can be any valid file name or valid directory path including a file name. In case **<path-file-name>** is deliminated by single quotes, it can contain any special characters allowed by the operating system in use. If the specified file exists, the user will be asked to replace it. This prompt is suppressed, if one of the follow three options, namely **append**, **replace**, or **genfilename**

has been set to **yes**. With the **append** option the output is appended to an existing file, whereas with the **replace** option the output possibly overwrites an existing file without further notice. If the **genfilename** option has been set, a file name of the form "**path-file-name-<num>**" will be generated, where **path-file-name** is the specified file name and where **<num>** is chosen as the smallest positive integer, such that the resulting file name does not exist yet. Thus, successive output commands with the options

```
file = myfile genfilename=yes
```

produce a series of files `myfile-1`, `myfile-2`, `myfile-3`, ... In particular, older files will never be overwritten.

1.3.3 Unix commands within SAMS

When using SAMS on a Unix operating system, the following additional commands are available from within SAMS.

!

Use the **!** command to execute a Unix command from within SAMS. In case several unix commands, separated by semicolons, are to be executed, they have to be enclosed within single-quotes. A temporary Unix shell can be opened by typing, for example, !sh or !bash. To exit the shell and return to SAMS just type exit or Ctrl-D.

Syntax:

```
!  <unix-command>;
!  '<unix-command>; <unix-command>; <unix-command>';
```

The syntax is equivalent to the syntax of the same command in PAUP*.

cd

Use the **cd** command to change the current working directory. If entered without arguments, the command reports the current working directory.

Syntax:

```
cd;
cd <new-directory>;
```

ls

Use the **ls** command to list the content of the current working directory. This command is simply an appreviation for **!ls**

Syntax:

```
ls [<options-for-ls>] [<file> ...];
```

1.3.4 The general SAMS commands

The following commands affect the general behavior of SAMS and its analysis methods.

Assume

Use the **assume** command to specify a predefined set of character positions that shall be excluded from the analysis (i.e. an exset). Exsets can be defined in the assumptions-block of a nexus-file.

Syntax:

```
assume [exset=<exset-name>];
```

This command implements part of the functionality of the **assume** command in PAUP*.

Delete

Use the **delete** command to exclude one taxon or a set of taxa from the analysis. The command name "delete" is a clear misnomer, since nothing is deleted. The name is used in order to be compatible with the commands used in PAUP*. "Deleted" taxa can always be "un-deleted", without having to load the data again, by using the **undelete** or **restore** command.

Syntax:

```
delete <set-of-taxa> [/only];
```

The <**set-of-taxa**> can be any set of valid taxon names or numbers specified in the NEXUS-syntax described in (Swofford 2003). If called with the **only** option, all taxa are restored before the taxa in <**set-of-taxa**> are deleted. Otherwise, the status of taxa not present in <**set-of-taxa**> remains unchanged.

The syntax is equivalent to the syntax of the same command in PAUP*.

Exclude

Use the **exclude** command to exclude one or a set of character positions from the analysis. Character positions can also be exclude by using the **assume** command or by specifying a default exclusion set (i.e. an exset) in an assumptions-block of a nexus file. Excluded characters can be re-included, without having to load the data again, by using

the **include** command.

Syntax:

```
exclude <set-of-character-positions> [/only];
```

The <**set-of-character-positions**> can be any set of valid position names or numbers specified in the NEXUS-syntax described in (Swofford 2003). If called with the **only** option, all positions are included before the positions in <**set-of-character-positions**> are excluded. Otherwise, the status of character positions not present in <**set-of-taxa**> remains unchanged.

The syntax is equivalent to the syntax of the same command in PAUP*.

Execute

Use the execute command to initiate that the specified nexus file is processed by SAMS.

Syntax:

```
execute <nexus file>;
```

The following nexus blocks can be interpreted by SAMS: Taxa-blocks, Character-blocks, Data-blocks, Assumptions-blocks, Tree-blocks, and SAMS-blocks. The contents of all other blocks are ignored. Commands in a SAMS-block are interpreted exactly in the same way as if they where entered on the command line, except that a colon is always required to terminate a command. The nexus file is interpreted until the "end of file" has been reached or the **quit** or **leave** command has been encountered within a SAMS-block.

Help

Use the **help** command to display a list of available SAMS-commands.

Syntax:

```
help;
```

Include

Use the **include** command to re-include character positions that have previously been excluded. Character positions can also be included with the assume command.

Syntax:

```
include <set-of-character-positions> [/only];
```

The <**set-of-character-positions**> can be any set of valid position names or numbers specified in the NEXUS-syntax described in (Swofford 2003). If called with the **only** option, all positions are excluded before the positions in <**set-of-character-positions**> are included. Otherwise, the status of character positions not present in <**set-of-taxa**> remains unchanged.

The syntax is equivalent to the syntax of the same command in PAUP*.

Log

Use the **Log** command to specify the name of the log file, and to open (i.e. start logging to) or close (i.e. stop logging to) the log file. As long as a log file is open, all output to the screen or to files is also written to the log file. It is not possible to open a new log file while another log file is already open.

Syntax:

```
log [ file = <filename> ]
    [ start = no|yes ]
    [ stop = no|yes ]
    [ append = no|yes ]
    [ replace = no|yes ]
    [ genfilename = no|yes ];
```

Description of options:

The **file=<filename>** option:
If a file name is specified with this option, this name replaces the old (or default) name of the log file. The name of the log file will also be changed if the current command does not open a log file, or if the attempt to open a file was not successful. The name of the log file stays in effect as long as it is not replaced with this option in the log command.

The **start= no|yes** option:
If the **start=yes** option has been specified, an attempt will be made to open a log file and start logging. The **start** option is automatically set to yes if the file option has been used successfully within the current log command. If no name for the log file has been set during the current SAMS-session, a default name will be used. In case a file with the current log file name exits, and if neither the **append**, **replace**, or **genfilename** option has been specified, the user will be asked whether to replace the existing file.

The **stop= no|yes** option:
If the **stop** option has been specified, the current log file will be closed and logging will be stopped.

The **append**, **replace**, and **genfilename** options have the same effect as described in section 1.3.2.

PrSequences

Use the **PrSequences** command to print the sequence data exactly in that form currently used in the analysis. That is, excluded positions or taxa are not printed. Furthermore, if gaps are currently treated as missing characters, they are displayed as missing characters. The data can be printed in the "Nexus", "PHYLIP", as well as in the "FASTA" format. In particular, this command can be used to convert data file that are available only in the Nexus-format to any of the aforementioned formats.

Syntax:

```
PrSequences [ file = <filename> ]
            [ append = no|yes ]
            [ replace = no|yes ]
            [ genfilename = no|yes ]
            [ format = nexus|phylip|fasta ];
```

Description of options:

The **file**, **append**, **replace**, and **genfilename** options are described in Section 1.3.2. With the **format** option it is possible to control the format used to display the data.

Restore and Undelete

Use the **restore** or the **undelete** command to re-include taxa that have previously been "deleted". Both commands have the same syntax and the same effect.

Syntax:

```
restore <set-of-taxa> [/only];
undelete <set-of-taxa> [/only];
```

The <**set-of-taxa**> can be any set of valid taxon names or numbers specified in the syntax described in (Swofford 2003). If called with the **only** option, all taxa are "deleted" before the taxa in <**set-of-taxa**> are undeleted. Otherwise, the status of taxa not present in <**set-of-taxa**> remains unchanged.

The syntax is equivalent to the syntax of the same commands in PAUP*.

SetGlobalParameters

Use the **SetGlobalParameters** command to set the global parameters of SAMS.

Syntax:

```
SetGlobalParameters [gapMode = newState | missing]
                    [consensusThreshold = real value in range [0..1]]
```

Description of parameters:

The **gapMode** parameter controls how gaps are treated in molecular sequence data. Note that this parameter effects the result of every analysis in SAMS, including the determination of base frequencies.

- newState: Gaps are treated as an additional state, i.e. as a fifth state in the case of DNA-data.
- missing: Gaps are treated as missing characters.

The value of the **consensusThreshold** parameter is relevant whenever a consensus sequence has to be computed for a set of taxa during an analysis. This is done as follows: For each site, the occurring characters are counted. If the most frequent character of a site occurs with a proportion larger than or equal to consensusThreshold, and if no other character occurs with this same proportion, this character is chosen as the consensus character. Otherwise the consensus character is set to the missing symbol.

Show

Use the **show** command to display the current status of the data that has been loaded into SAMS.

Syntax:

```
show [ file = <filename> ]
     [ append = no|yes ]
     [ replace = no|yes ]
     [ genfilename = no|yes ];
```

Description of options:

The **file**, **append**, **replace**, and **genfilename** options are described in Section 1.3.2.

ShowGlobalParameters

Use the **ShowGlobalParameters** command to display a list of all global parameters and their current value.

Syntax:

```
ShowGlobalParameters
```

Undelete

For a description see the **restore** command above.

1.3.5 General analysis commands

PrBaseFrequncies

Use the **PrBaseFrequncies** command to display the base frequencies of the currently active data, i.e. of the taxa and character positions that are not deleted or excluded.

Syntax:

```
PrBaseFrequncies [ file = <filename> ]
                 [ append = no|yes ]
                 [ replace = no|yes ]
                 [ genfilename = no|yes ]
                 [ format = relFrequencies | absFrequencies |
                            relFrequenciesOnlyAT | relFrequenciesOnlyCG ];
```

Description of options:

The **file**, **append**, **replace**, and **genfilename** options are described in Section 1.3.2.

1.3.6 Commands in the PHYSID module of SAMS

This module provides a set of analysis routines to determine character positions that support splits. The data analysis algorithms in the PHYSID module are described in detail in Section 2.1.

PhysidBootstrap

Use the **PhysidBootstrap** command to perform a bootstrap analysis in order to estimate the variation of the support values for the splits with high support that were found in a preceding call to the **PhysidMultiSplitsDetSupPos** command. The estimated variations are then used to determine error-bars for the corresponding split support values.

After having determined support values for putative splits in an alignment, the question arises how certain these values are from a statistical point of view. To be more

precise, what is the expected variation of these support values if we would have drawn the positions in the given data set from much longer sequences or from several independent data sets which all evolved according to the same true model of evolution. Unfortunately, this true “universal set” of site patterns in the alignment is inaccessible for us and the only chance we have is to estimate the variation of the support values from a *population of data sets* constructed from the given data set itself. Statistical methods of this kind are known as bootstrap methods.

There are two major approaches in phylogenetic studies to obtain a population of new data sets from an original data set: The first involves re-sampling new data sets directly from the original data set, which is known as a non-parametric bootstrap analysis. The second involves determining the most probable model of evolution and phylogenetic tree in order to compute simulate data sets, which is known as parametric bootstrap analysis. Both approaches can be used within SAMS, where in case of the second approach a file with the simulated data sets has to be supplied.

Syntax:

```
PrBaseFrequncies [ dataFile = <file-name> ]
                 [ nReps = positiv integer ]
                 [ seed = odd positive integer ]
                 [ nBest = positive integer ]
                 [ MinTaxaInIgOrOg = positive integer ];
```

If a **dataFile** is specified, this file has to contain a *population of data sets*, of which the original data file is expected to be a good representative. The population of data sets is used to estimate the variance of support values of splits. The number of replicates is determined by the number of data sets present in the file. If **dataFile**is not specified, a non-parametric bootstrap is performed in which a *population of data sets* is constructed by re-sampling (i.e. randomly drawing positions of) the original data set.

The **nReps** and **seed** option are only relevant in the case of a non-parametric bootstrap analysis, i.e. if no dataFile is specified. **nReps** is the number of bootstrap replicates that shall be computed; the default value is 100. **seed** is the seed value that initialises the pseudo-random generator used to computes the re-sampled data-sets; a default value is obtained form the system clock. A seed should be specified if a reproducible bootstrap analysis is intended.

Since a bootstrap analysis is computationally intense, the variation of support values can only be determined for a limited number of splits. The splits that are analysed are those which where found to have highest support values in a preceeding call to the **PhysidMultiSplitsDetSupPos** command. (As a consequence the **PhysidBootstrap** command can only be used after **PhysidMultiSplitsDetSupPos** has been called for the given data set.) The number of splits that are analysed in this way can be specified

with the **nBest** option. Its default value is 100. Furthermore it is possible to restrict the analysis to splits with a certain minimum number of taxa in the in- or outgroup specified by **MinTaxaInIgOrOg**.

PrPhysidBootstrap

Use this command to print the results of the last call to the **PhysidBootstrap** command.

Syntax:

```
PrPhysidBootstrap [ ConfidenceLimit = real number in range 0..1 ]
                  [ printSpectrumTable = yes|no ]
                  [ printTableTitle = yes|no ]
                  [ printBinarySplitNumber = yes|no ]
                  [ printTaxaList = yes|no ]
                  [ printParameterValues = yes|no ]
                  [ printBootstrapComparissonMatrix = yes|no ];
```

After the **PhysidBootstrap** command has been used to determined the full distribution of support values of splits among a population of data set, a call to the **PrPhysidBootstrap** command gives the following output: For each split, the distribution of the total support values as well as the distribution of the support values for the outgroup and the ingroup are analysed separately. For each distribution the *minimum*, a *lower* limit, the mean, an *upper* limit and the *maximum* are printed. The *lower* limit and the *upper* limit are determined according to the specified **ConfidenceLimit** such that centered to the mean of the distribution a proportion of **ConfidenceLimit** support values are found in this range. Thus, for values of **ConfidenceLimit=0.95**, the default value, only 5% of the support values in the population of data sets lie outside the range from *lower* limit to *upper* limit.

The **printSpectrumTable**, **printTableTitle**, **printBinarySplitNumber**, **printTaxaList**, **printParameterValues** and **printBootstrapComparissonMatrix** options allow the user to specify which information will be displayed with the call to this command. The default is to print all information except the list of taxa.

- **printSpectrumTable=yes|no**: Print table containing the support values of splits.
- **printTableTitle=yes|no**: Print a table title.
- **printBinarySplitNumber=yes|no**: Print a list of 0's and 1's indicates whether taxa occur in the in- or outgroup of the corresponding split. For example a 1 at the third position indicates the occurrence of taxon 3 in the ingroup, whereas a 0 indicates its occurence in the outgroup.

- **printTaxaList=yes|no**: Print a list of the taxa in the ingroup and outgroup of the splits occuring in the table of splits with highest support.
- **printParameterValues=yes|no**: Print values of the parameter relevant for the current analysis.
- **printBootstrapComparissonMatrix=yes|no**: Print a matrix in which each element (i, j) is the number of bootstrap replicates for which that split i has a higher support than split j.

PhysidDetSupPos

Use the **PhysidDetSupPos** command to determine the supporting positions for a specific split. The command does not result in any output. To view the determined supporting positions use the **PrPhysidSupPos** command.

Syntax:

```
PhysidDetSupPos <set-of-taxa>;
```

The <set-of-taxa> can be any set of taxa specified in the NEXUS-syntax described in (Swofford 2003). This set defines the ingroup of the split that is to be analysed.

PhysidMultiSplitsDetSupPos

Use the **PhysidMultiSplitsDetSupPos** command to determine the number of supporting character positions for "specific sets" of splits. The support for the splits that have been analysed with this command can be displayed in tabular form with the aid of the **PrPhysidMultiSplitsSupPos** command.

Syntax:

```
PhysidMultiSplitsDetSupPos [ splits = occurring | all | search ]
                           [ searchDepth = positive integer ]
                           [ searchProportion = real number in range 0..1 ]
                           [ supportThreshold = positive integer ];
```

Description of options:

If the number of taxa, here denoted by n, is large, it becomes impossible to determine supporting positions for all 2^n possible splits. With the **splits** options, it is possible to specify the set of splits that are to be analysed.

- **splits=occurring** (default): Only the splits that occur in the data are analysed.
- **splits=all:** All possible 2^n splits are analysed. Since the number of splits increases exponentially with the number of taxa, this option is only allowed if $n \leq 15$.

- **splits=search:** In a first step, all splits are analysed which occur in the data. In a second step, a specified proportion of the best occurring splits is used as starting points for a search, in which taxa are added and removed from the in-groups of splits, with the aim of finding splits not explicitly occurring in the data, but having a *high number* of supporting positions.

The following three options are only relevant if **splits=search** has been specified. They control the search depth, the number of best splits used as starting points in the search, and a threshold value for the number of supporting character positions above which we are interested in a new split:

The **searchDepth**, which has a default value of 2, has the following meaning: For each split chosen as a “starting point”, we systematically add and remove taxa in the in-group, such that for each variation, the in-group of a split is allowed to differ from its original by at most “searchDepth” taxa. All splits which are found in this *neighborhood* of a “starting point split” are analysed.

With **supportThreshold** it is possible to specify the support value above which a split will be used as a new starting point above which it will be output. The default value is the minimum of the support of the 120 occuring splits with highest support.

With **searchNum** it is possible to specify the number of occurring splits that are used as starting points of the search. The default value depends on the number of splits that occur in the data set. It is 20% of the occuring splits with a minimum value of 200, but off course not larger than the number of splits occuring in the data set.

Note that the default values of **searchDepth**, **searchNum**, and **supportThreshold** are chosen such that they result in a thorough search, with the consequence that the search can sometimes, especially for larger and/or uninformative data set, take in the order of several hours. The preferable possibility to reduce computation time is to first compute the support values without search and to adjust the **supportThreshold** to a value probably only slightly larger than the default value.

PrPhysidSupPos

Use this command to print the results of the last call to the **PhysidDetSupPos** command.

Syntax:

```
PrPhysidSupPos [ format = asSummary | asSeq | asSeqInterleave ]
               [ file = <filename> ]
               [ append = no|yes ]
               [ replace = no|yes ]
               [ genfilename = no|yes ];
```

Description of options:
The **file**, **append**, **replace**, and **genfilename** options are described in Section 1.3.2.
The **format** options control how information about supporting positions is displayed:

- **format=asSummary** (default): The numbers (Anzahl) of character positions that support the in- and outgroup of the split are displayed. Different degrees of support are distinguished, which is described in detail in Section 2.5. Furthermore, a list of all position numbers of supporting positions is printed.
- **format=AsSeq:** The sequence data for the supporting positions is printed, such that for each taxon all characters are printed on one line. Ingroup and outgroup taxa are printed in blocks separated by a line.
- **format=AsSeqInterleave:** The sequence data for the supporting positions is printed, such that for each taxon at most 50 character positions will be printed on one line. Ingroup and outgroup taxa are printed in blocks separated by a line.

PrPhysidMultiSplitsSupPos

Use the **PrPhysidMultiSplitsSupPos** command to print the results of the last call to the **PhysidMultiSplitsDetSupPos** command.

Syntax:

```
PrPhysidMultiSplitsSupPos [ file = <filename> ]
                          [ append = no|yes ]
                          [ replace = no|yes ]
                          [ genfilename = no|yes ]
                          [ nbest = positive integer ]
                          [ minTaxaInIgOrOg = positive integer ]
                          [ printSpectrumTable = yes|no ]
                          [ printTableTitle = yes|no ]
                          [ printBinarySplitNumber = yes|no ]
                          [ printTaxaList = yes|no ]
                          [ printParameterValues = yes|no ];
```

Description of options:

The **file**, **append**, **replace**, and **genfilename** options are described in Section 1.3.2.

With the **nbest** option it is possible to specify the maximum number of splits that are to be printed with this command. Output starts with the split of highest support, and not more than **nbest** splits will be displayed, even if more splits have been analysed. The value of **nbest** must be an integer in the range $[0, 4294967296]$. To ensure that all splits are printed, simply specify a sufficiently high number. The default value is 4294967296.

With the **minTaxaInIgIrOg** option it is possible to control the minimum number of taxa that a split must have in the in-group and in the out-group in order for the split to be printed with this command. The default value of **minTaxaInIgIrOg** is 1.

The **printSpectrumTable**, **printTableTitle**, **printBinarySplitNumber**, **printTaxaList**, and **printParameterValues** options allow the user to specify which information will be displayed with the call to this command. The default is to print all information.

- **printSpectrumTable=yes|no**: Print table containing the support values of splits.
- **printTableTitle=yes|no**: Print a table title.
- **printBinarySplitNumber=yes|no**: Print a list of 0's and 1's indicates whether taxa occur in the in- or outgroup of the corresponding split. For example a 1 at the third position indicates the occurrence of taxon 3 in the ingroup, whereas a 0 indicates its occurence in the outgroup.
- **printTaxaList=yes|no**: Print a list of the taxa in the ingroup and outgroup of

the splits occuring in the table of splits with highest support.

- **printParameterValues=yes|no**: Print values of the parameter relevant for the current analysis.

These options are useful to suppress certain parts of the usual output and/or to direct different parts of the output to different files.

SetPhysidParameters

Use the **SetPhysidParameters** command to set the parameters of the physid-module of SAMS.

Syntax:

```
SetPhysidParameters [ MaxIgNoise=<value> ] [ MaxOgNoise=<value> ]
                    [ MaxSimIgToOg=<value> ] [ MaxSimOgToIg=<value> ]
                    [ sequenceComparison = pairwise | consensus ];
```

Description of parameters:

The numerical parameters **MaxIgNoise**, **MaxOgNoise**, **MaxSimIgToOg**, as well as **MaxSimOgToIg** can take on any real value in the range [0..1]. The usage and effect of these parameters is described in detail in Section 2.2.

ShowPhysidParameters

Use the **ShowPhysidParameters** command to view all parameter settings in the physid-module of SAMS.

Syntax:

```
ShowPhysidParameters;
```

References

Maddison D.R., Swofford D.L., Maddison W.P., 1997, NEXUS: an extensible file format for systematic information. Syst Biol. **46** (4), p.590-621.

Mayer, C. and Wägele, J.W., 2006, (in preparation).

Swofford, D. L., 2003, PAUP*. Phylogenetic Analysis Using Parsimony (*and Other Methods). Version 4. Sinauer Associates, Sunderland, Massachusetts.

Wägele, J.W. and Röding F., 1998, A Priori Estimation of Phylogenetic Information Conserved in Aligned Sequences, Molecular Phylogenetics and Evolution, **9** (3), pp. 358-365.
